# Comparing high versus low-altitude populations to test human adaptations for increased ventilation during sustained aerobic activity

**DOI:** 10.1038/s41598-022-13263-5

**Published:** 2022-07-01

**Authors:** W. Éamon Callison, Melisa Kiyamu, Francisco C. Villafuerte, Tom D. Brutsaert, Daniel E. Lieberman

**Affiliations:** 1 Department of Human Evolutionary Biology, Harvard University, Cambridge, Massachusetts USA; 2 Laboratorio de Fisiología del Transporte de Oxigeno, Universidad Peruana Cayetano Heredia, Lima, Peru; 3 Department of Exercise Science, Syracuse University, Syracuse, New York USA

**Keywords:** Biological anthropology, Anatomy, Respiration

## Abstract

Despite aerobic activity requiring up to tenfold increases in air intake, human populations in high-altitude hypoxic environments can sustain high levels of endurance physical activity. While these populations generally have relatively larger chest and lung volumes, how thoracic motions actively increase ventilation is unknown. Here we show that rib movements, in conjunction with chest shape, contribute to ventilation by assessing how adulthood acclimatization, developmental adaptation, and population-level adaptation to high-altitude affect sustained aerobic activity. We measured tidal volume, heart rate, and rib-motion during walking and running in lowland individuals from Boston (~ 35 m) and in Quechua populations born and living at sea-level (~ 150 m) and at high altitude (> 4000 m) in Peru. We found that Quechua participants, regardless of birth or testing altitudes, increase thoracic volume 2.0–2.2 times more than lowland participants (p < 0.05). Further, Quechua individuals from hypoxic environments have deeper chests resulting in 1.3 times greater increases in thoracic ventilation compared to age-matched, sea-level Quechua (p < 0.05). Thus, increased thoracic ventilation derives from a combination of acclimatization, developmental adaptation, and population-level adaptation to aerobic demand in different oxygen environments, demonstrating that ventilatory demand due to environment and activity has helped shape the form and function of the human thorax.

## Introduction

**Figure 1:**
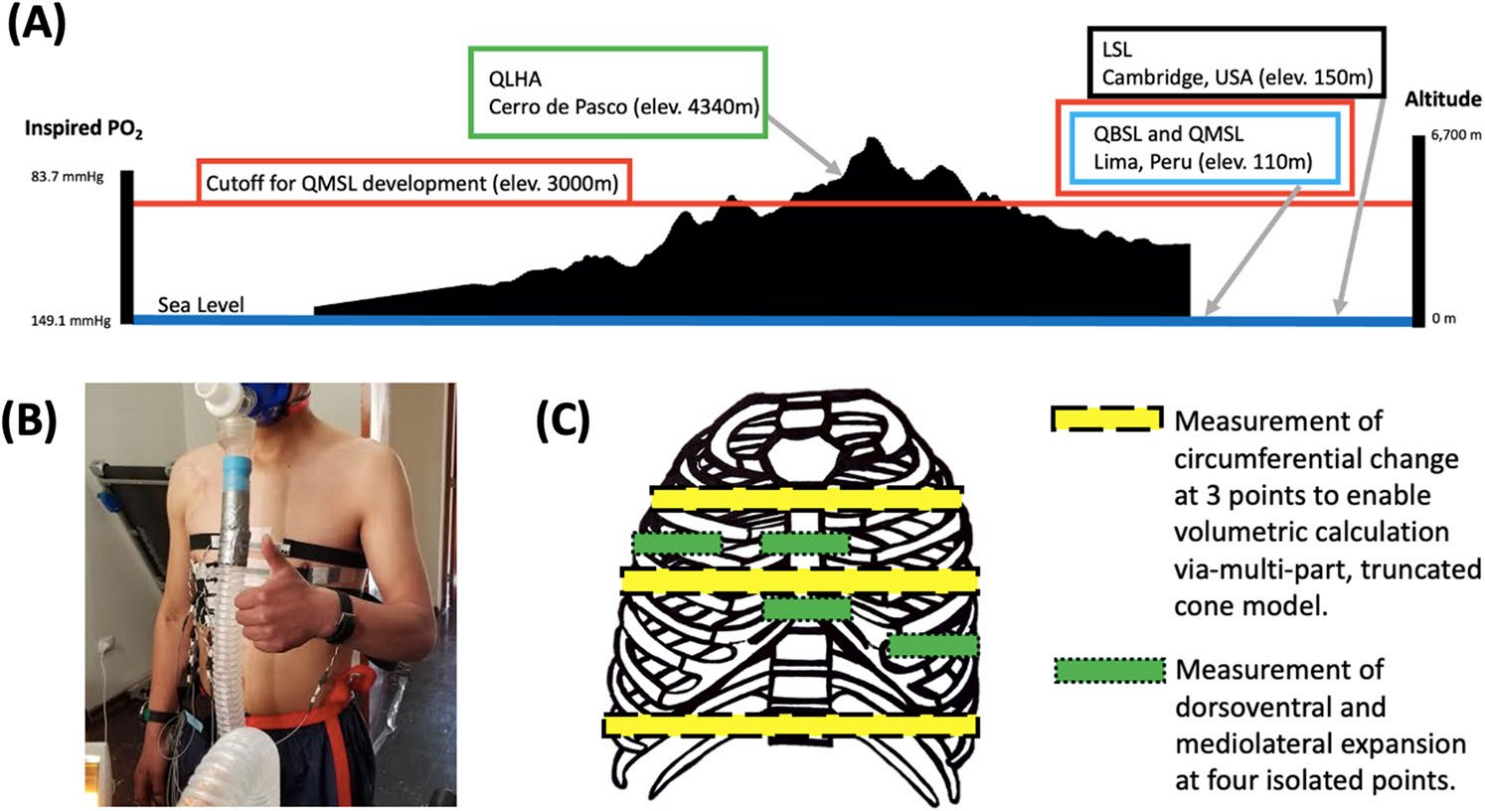
(**A**) The partial pressure of inspired oxygen decreases with elevation. Participant populations were from different oxygen environments and experiments took place at different altitudes. (**B**) A participant outfitted with our nanocomposite devices, mask, and spirometer. (**C**) Approximate device placement on human participants (ventral view) corresponding to thoracic circumference measurements.

Unlike other apes, humans are capable of sustained moderate and vigorous-intensity physical activities (PA) thanks to a suite of skeletal, muscular, thermoregulatory, cardiovascular, and metabolic adaptations^1–4^. Resting mass-specific minute ventilation (${\dot{\text{V}}}$_E_) in humans averages approximately 0.1 L/min/kg, but vigorous aerobic PA, like running, requires sustained breathing rates of as much as 1.3–2.5 L/min/kg^5,6^. Consequently, an important adaptation for aerobic activity is the ability to increase maximal oxygen uptake (${\dot{\text{V}}}$O_2MAX_) by augmenting aspects of ventilatory capacity, including the volume of air inspired per breath (tidal volume; V_T_) and per minute (${\dot{\text{V}}}$_E_)^6,7^. Because these demands are even greater at high altitude (Fig. 1A), there has been strong selection for high-altitude populations to facilitate aerobic PA in hypoxic environments by increasing ${\dot{\text{V}}}$O_2MAX_^8^. For example, Inca *chaskis* (messengers) were able to run 10–15 km at ~ 10 km/h through rugged Andean terrain^9^.

**Figure 2:**
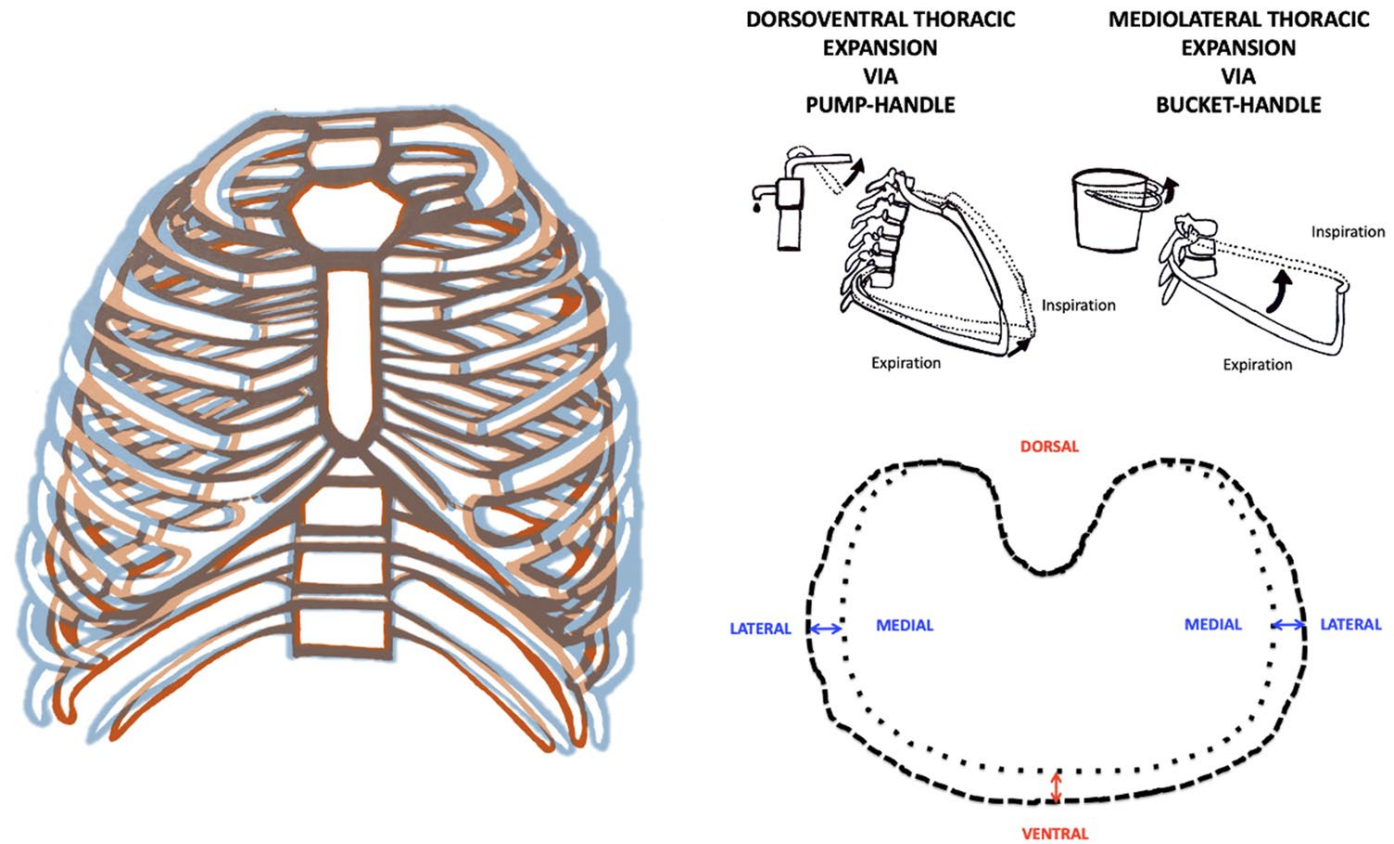
The thorax increases in volume during ventilation due to the action of the ribs through the pump-handle and bucket-handle motions. Changes in the maximum circumference of the thorax during ventilation result from the pump-handle (dorsoventral; red) and bucket-handle (mediolateral; blue) rib motions in humans.

Individual and population-level adaptations to high altitude have previously been shown to alter physiology^10–20^ and increase respiratory volumes^21–30^, chest dimensions^28,31–36^ and ${\dot{\text{V}}}$O_2MAX_^8,16,37,38^. However, even though thoracic expansion can be predicted from a biomechanical model (Figs. S1 and S2) based on dorsoventral (pump-handle; PH) and mediolateral (bucket-handle; BH) rib movements (Fig. 2), there has been little research on variations in thoracic ventilatory biomechanics despite the importance of chest expansion and contraction for inhalation and exhalation.

Evidence that the human chest evolved to augment ventilation during PA^4^ suggests that thoracic expansion may be increased in humans living at high altitude. Because acclimatization, development, and population-level adaptations influence breathing in different oxygen environments^16^, this study takes advantage of a natural experiment^16,37–39^ to measure thoracic contributions to ventilation during activity among low- versus high-altitude dwelling individuals from the Quechua people and from a sample of sea-level dwellers from the greater Boston area (Fig. 1A). Using population-level comparisons, we test the general hypothesis that increased ventilatory demand selected for increased thoracic ventilation. We also test three specific hypotheses concerning the basis for these adaptations^40,41^:

1. Adulthood Acclimatization Hypothesis: Individuals living in hypoxic high-altitude environments use more thoracic motion to breathe than individuals from the same population acclimatized to low altitude. We test differences in acclimatization by comparing Quechua living at high altitude (QLHA) in Cerro de Paco, Peru to Quechua born at high altitude who have migrated to sea level (QMSL) in Lima, Peru.
2. Developmental Adaptation Hypothesis: Individuals developmentally adapted to high-altitude environments have increased chest dimensions and rib movements relative to members of the same population who developed at low altitude, thereby increasing chest expansion during ventilation. We test differences in high-altitude influenced developmental adaptation by comparing QMSL participants to Quechua born at sea level (QBSL).
3. Population-Level Adaptation Hypothesis: Individuals from high-altitude populations use more thoracic motion to breathe than individuals from low-altitude populations, even when breathing at sea level, because of population-level increases in thoracic dimensions and thoracic motion. We test differences in population-level adaptation by comparing high altitude-adapted QBSL participants to lowland participants living at sea level in Boston (LSL).

## Results

### Participants and anthropometrics

QBSL participants, QMSL participants, QLHA participants, and LSL participants did not differ significantly in age. Stature was not different between Quechua populations, though Quechua participants were significantly shorter on average than LSL participants (Table 1). QLHA and QMSL participants had smaller body masses compared to QBSL and LSL participants (Table 1). QBSL and LSL participants had shallower chests than QMSL and QLHA participants, and LSL chests were less broad than those from the Quechua populations (Table 1 and Table S1). However, LSL participants had significantly larger chest volumes, as expected based on height and body mass (Table 1). QMSL participants had larger unadjusted forced vital capacity (FVC) and forced expiratory volume (FEV_1_) values relative to QBSL participants, similar to a previous study with similar groups^37^.

**Table 1:** Participant anthropometrics, with values given as mean ± SD. NS: not significant; *: p ≤ 0.05; **: p ≤ 0.01; ***: p ≤ 0.001.

	QLHA (n = 20)	QMSL (n = 17)	QBSL (n = 16)	LSL (n = 15)
Age (years)	23.15 (± 3.23)^NS(QMSL),NS(QBSL),NS(LSL)^	24.29 (± 3.27)^NS(QHLA),NS(QBSL),NS(LSL)^	24.06 (± 3.40)^NS(QHLA),NS(QMSL),NS(LSL)^	22.73 (± 4.01)^NS(QHLA), NS(QMSL),NS(QBSL)^
Standing height (cm)	164.78 (± 5.82)^NS(QMSL),NS(QBSL),^***^(LSL)^	165.57 (± 5.01)^NS(QLHA),NS(QBSL),^***^(LSL)^	166.09 (± 5.49)^NS(QLHA),NS(QMSL),^***^(LSL)^	178.93 (± 5.65)***^(QLHA),^***^(QMSL),^***^(QBSL)^
Body mass (kg)	64.94 (± 10.61)^NS(QMSL),^^NS^^(QBSL),^*^(LSL)^	67.97 (± 8.10)^NS(QLHA),^^NS^^(QBSL),^^NS^^(LSL)^	70.83 (± 9.98)^NS^^(QLHA),^^NS^^(QMSL),NS(LSL)^	72.51 (± 8.51)*^(QLHA),^^NS^^(QMSL),NS(QBSL)^
Sitting height (cm)	88.68 (± 2.82)^NS(QMSL),NS(QBSL),^***^(LSL)^	88.41 (± 2.16)^NS(QHLA),NS(QBSL),^***^(LSL)^	89.36 (± 3.55)^NS(QHLA),NS(QMSL),^**^(LSL)^	94.12 (± 2.97)***^(QLHA),^***^(QMSL),^**^(QBSL)^
BMI	23.89 (± 3.47)^NS^^(QMSL),^^NS^^(QBSL),^^NS^^(LSL)^	24.79 (± 2.72)^NS^^(QLHA),NS(QBSL),^*^(LSL)^	25.67 (± 3.22)^NS^^(QLHA),NS(QMSL),^*^(LSL)^	22.70 (± 3.04)^NS^^(QLHA),^*^(QMSL),^*^(QBSL)^
Resting chest depth (cm)	24.91 (± 4.10)^NS(QMSL),^^NS^^(QBSL),^^NS^^(LSL)^	25.56 (± 1.64)^NS(QLHA),^*^(QBSL),^*^(LSL)^	23.84 (± 2.83)^NS^^(QLHA),^*^(QMSL), NS(LSL)^	23.89 (± 1.72)^NS^^(QLHA),^*^(QMSL),NS(QBSL)^
Resting middle chest width (cm)	31.20 (± 1.55)^NS(QMSL),NS(QBSL),^^NS^^(LSL)^	31.00 (± 3.18)^NS(QLHA),NS(QBSL),NS(LSL)^	32.14 (± 2.79)^NS(QLHA),NS(QMSL),NS(LSL)^	30.87 (± 2.04)^NS^^(QLHA),NS(QMSL),NS(QBSL)^
Resting chest volume (L)	11.05 (± 2.71)^NS(QMSL),NS(QBSL),^***^(LSL)^	11.17 (± 2.17)^NS(QLHA),NS(QBSL),^***^(LSL)^	12.53 (± 2.90)^NS(QLHA),NS(QMSL),^***^(LSL)^	17.44 (± 2.36)***^(QLHA),^^***(QMSL),***(QBSL)^

### Change in mass-specific tidal volume, respiration rate and mass-specific minute ventilation with heart rate

Mass-specific V_T_ (L/kg-BTPS; body temperature and pressure, saturated; Fig. S3) and respiration rate (f_R_; Fig. S4A) increased with heart rate ($f_{H,scope} = \frac{{f_{H,measured} - f_{H, resting} }}{{f_{H,max} - f_{H,resting} }} \times 100$) across all populations. Mass-specific ${\dot{\text{V}}}$_E_ (L/kg/min-BTPS) also increased with f_H,scope_ in all populations due to larger V_T_ and faster f_R_ during walking and running (Fig. S4B). However, LSL and QLHA participants increased mass-specific ${\dot{\text{V}}}$_E_ (L/kg/min-BTPS) 1.2–1.4 times more than in Quechua populations living at sea level (Fig. S4B).

### Absolute change in thoracic volume with tidal volume

**Figure 3:**
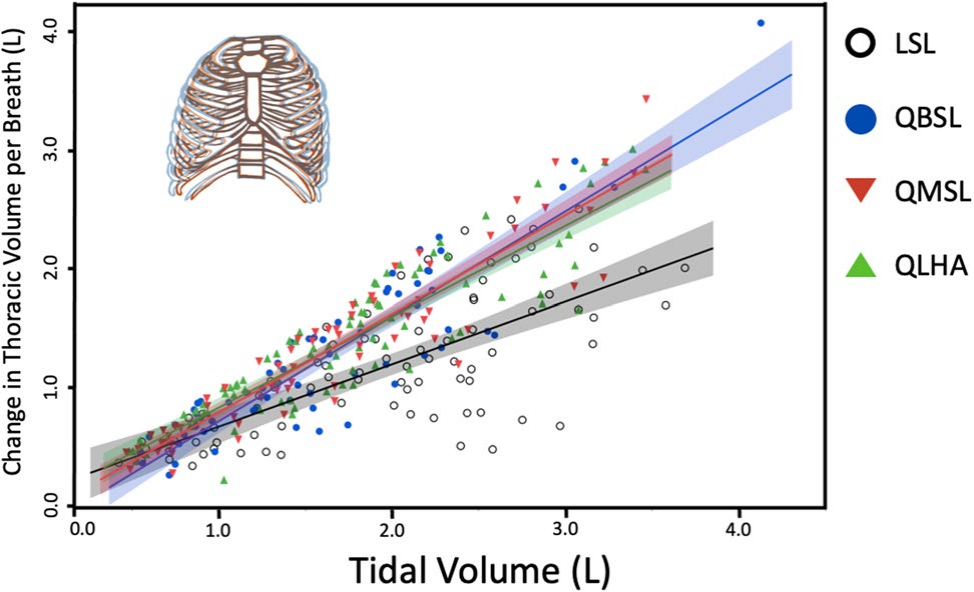
Absolute thoracic expansion and contraction (L) increased with absolute V_T_ (L) significantly more in QLHA (slope ± S. 0.75 ± 0.03, R^2^ = 0.81; p = 0.002), QMSL (0.81 ± 0.04, R^2^ = 0.87; p < 0.001) and QBSL (0.83 ± 0.06, R^2^ = 0.82; p < 0.001) than in LSL (0.57 ± 0.05, R^2^ = 0.52) participants. Shading represents 95% CIs.

We measured thoracic movement and V_T_ over a range of f_H_. The absolute change in thoracic volume per breath (L) increased with absolute V_T_ (L-BTPS) in all four populations (Fig. 3). However, despite having smaller absolute chest volumes (Table 1), change in thoracic volume per breath (L) rose 1.3–1.5 times more in Quechua populations than in LSL participants (p < 0.01; Fig. 3). The relative contribution of the thorax to V_T_ remained nearly constant across f_H_ in all populations, with no significant differences between observed between LSL and Quechua participants (Fig. S5A). However, when active, Quechua individuals appear to generally use the chest to breathe more than LSL participants (Fig. S5A), despite using the diaphragm to ventilate more during FVC (Fig. S5B).

### Change in thoracic volume and thoracic ventilation during physical activity normalized to body mass and chest size

**Figure 4:**
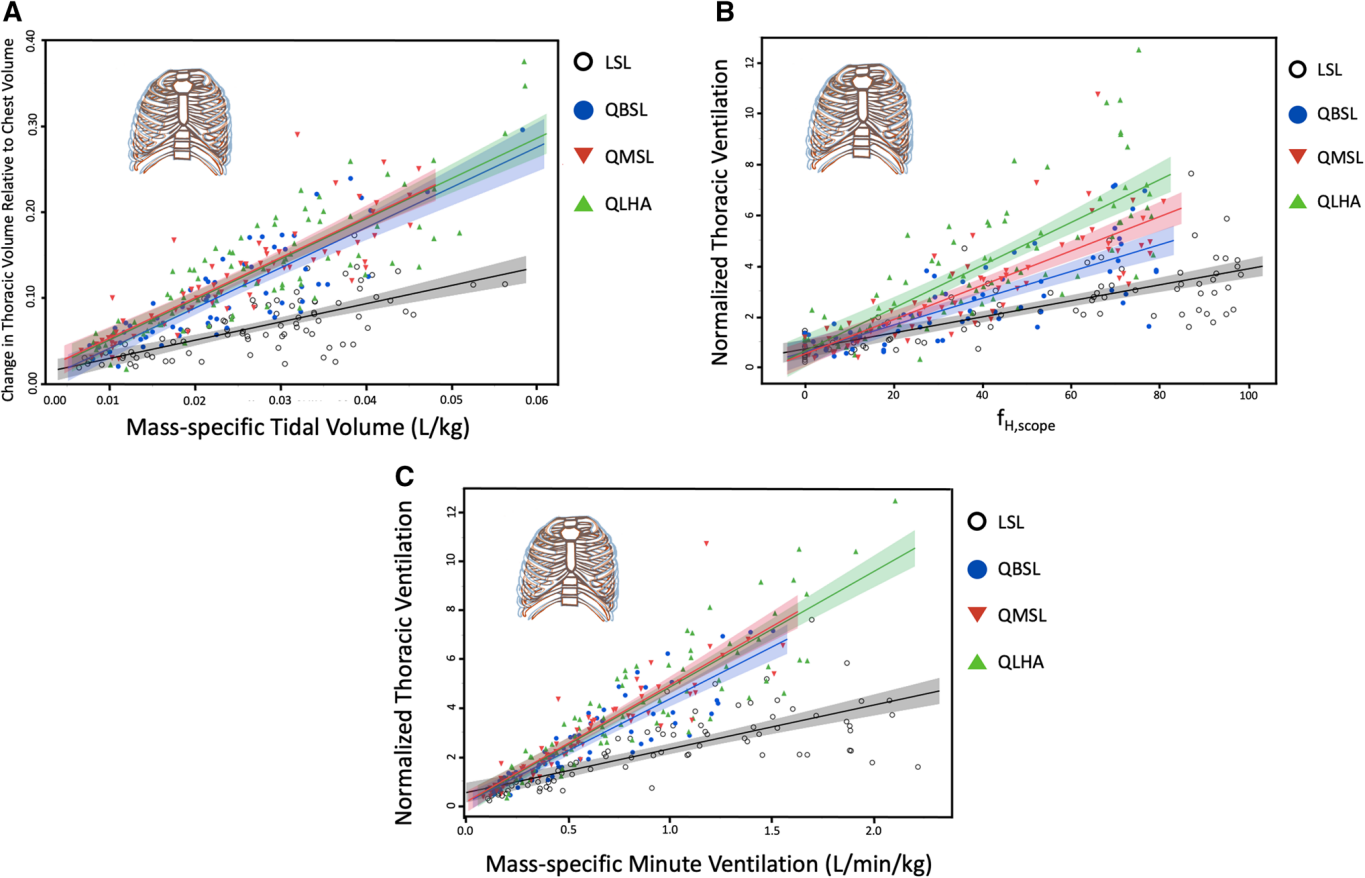
(**A**) Thoracic expansion and contraction relative to chest volume increased with mass-specific V_T_ (L/kg) in QLHA (slope ± S. 4.49 ± 0.23, R^2^ = 0.47), QMSL (4.84 ± 0.24, R^2^ = 0.66), QBSL (4.51 ± 0.24, R^2^ = 0.50), and LSL (2.27 ± 0.17, R^2^ = 0.36) participants. Shading represents 95% CIs. (**B**) Normalized thoracic ventilation, or the amount air inhaled per minute using the chest normalized to average chest volume, increases with f_H,scope_ in all populations. (**C**) Normalized thoracic ventilation increases with mass-specific ${\dot{\text{V}}}$_E_ (L/min/kg-BTPS) in QLHA (slope ± S. 4.74 ± 0.19, R^2^ = 0.80), QMSL (4.94 ± 0.23, R^2^ = 0.78), QBSL (4.18 ± 0.22, R^2^ = 0.78) and LSL (2.26 ± 0.15, R^2^ = 0.55) participants. Shading represents 95% CIs. Multiple measures from individual participants are presented together for graphical clarity. Repeated measures in participants are accounted for and fixed effects assessed via non-linear mixed effects models using generalized least squares (see “Statistical analysis”).

To test whether Quechua populations use thoracic movements relatively more than lowlanders to increase V_T_ during PA, we measured thoracic expansion and contraction, V_T_ and ${\dot{\text{V}}}$_E_ over a range of f_H_ (Fig. 4). Because mammalian lung volume scales isometrically with body mass^42^, V_T_ was normalized by each participant’s body mass. Changes in thoracic volume were measured as the difference in thoracic volume between inspiration and expiration, and the change in thoracic volume per breath was then normalized to the average volume of the chest for each trial and calculated as percent changes to allow inter-individual comparisons between participants with different sized chests.

The change in thoracic volume per breath relative to thoracic volume increased with mass-specific V_T_ (L/kg-BTPS) in all four populations (Fig. 4). However, normalized change in thoracic volume per breath rose 2.0–2.2 times more in Quechua populations than in LSL participants (p < 0.001; Fig. 4A). Significant differences between Quechua populations were not observed. Correspondingly, when f_R_ was taken into account, thoracic ventilation normalized to average chest volume ($Normalized\, Thoracic \,Ventilation = \frac{Change\, in\, Thoracic\,Volume\, per\, Breath}{{Average\, Volume\, of\, the\, Chest}} \times f_{R}$) also increased with f_H,scope_ in all participants (Fig. 4B), though normalized thoracic ventilation increased 1.5–2.4 times more in Quechua participants than in LSL participants with higher f_H_ (p < 0.001). Finally, normalized thoracic ventilation rose with mass-specific ${\dot{\text{V}}}$_E_ (L/kg/min-BTPS) in all populations (Fig. 4C). Again, normalized thoracic ventilation increased 1.9–2.2 times more in Quechua participants than in LSL participants (p < 0.001) with greater mass-specific ${\dot{\text{V}}}$_E_ (L/min/kg).

### Change in thoracic ventilation corresponding to atmospheric oxygen concentration

**Figure 5:**
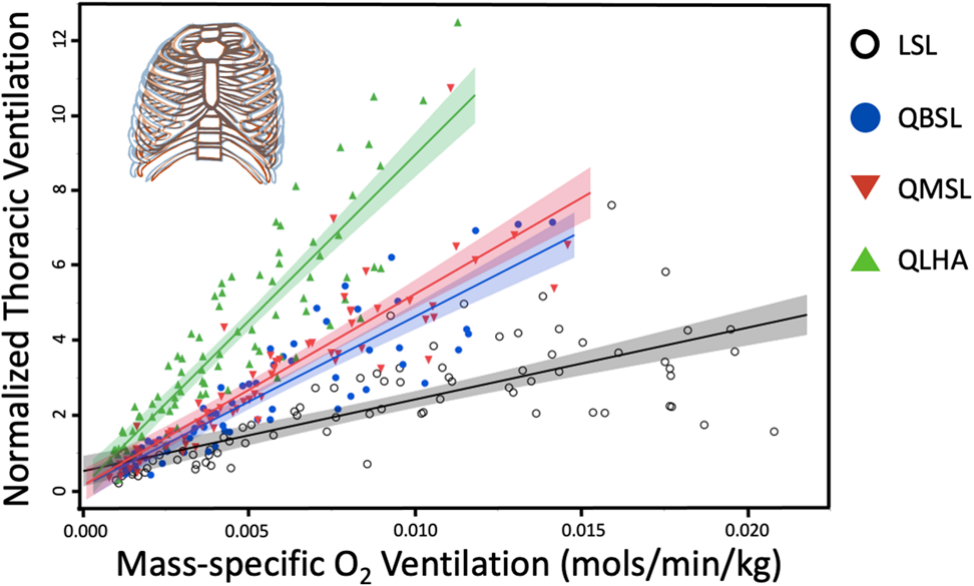
Normalized thoracic ventilation (the amount air inhaled per minute using the chest normalized to average chest volume) increases with mass-specific O_2_ ventilation (moles/min/kg) in QLHA participants (slope ± S. 885.01 ± 34.82, R^2^ = 0.80), QMSL (527.98 ± 24.99, R^2^ = 0.78), QBSL (445.90 ± 22.99, R^2^ = 0.78) and LSL participants (241.23 ± 16.05, R^2^ = 0.55). Shading represents 95% CIs. Multiple measures from individual participants are presented together for graphical clarity. Repeated measures in participants are accounted for and fixed effects assessed via non-linear mixed effects models using generalized least squares (see “Statistical analysis”).

Because every breath contains fewer oxygen molecules at altitude relative to sea level, we also corrected V_T_ by PO_2_ to reflect the moles of the oxygen inhaled per minute (mass-specific O_2_ ventilation; moles/kg/min-BTPS; Fig. 5). This correction permits us to test how thoracic function responds to the amount of oxygen present. QLHA participants increased normalized thoracic ventilation approximately 1.7–2.0 times more than QMSL (p < 0.001) and QBSL (p < 0.001) participants with increasing mass-specific O_2_ ventilation (moles/kg/min). QMSL and QBSL participants increased normalized thoracic ventilation approximately 1.9–2.2 times more than LSL participants (p < 0.001). Finally, QLHA participants increased normalized thoracic ventilation 3.7 times more than LSL participants (p < 0.001) with increasing mass-specific O_2_ ventilation (moles/kg/min).

### Thoracic volume change at rest

We observed differences in resting thoracic volume change relative to average chest volume between QLHA participants and QMSL (p = 0.034) and QBSL (p = 0.075) participants (Fig. S6). The largest resting difference was observed between LSL and QLHA (p = 0.002) participants. Overall, Quechua populations had 1.4–1.9 times greater resting thoracic expansions and contractions than lowlanders at sea level.

### Change in dorsoventral and mediolateral expansions with increasing heart rate

To test if specific rib movements, in conjunction with observed population differences in chest dimensions, affect thoracic function during PA, we measured PH and BH rib motions in participants to compare dorsoventral and mediolateral thoracic expansion. Dorsoventral (PH) and mediolateral (BH) thoracic expansion increased with f_H_ in all populations (Fig. S7). In the PH mechanism, dorsoventral expansion relative to chest depth increased 1.3–1.6 times more in QLHA (p = 0.037), QMSL (p < 0.001) and QBSL (p < 0.001) participants than in LSL participants. In the BH mechanism, change in mediolateral expansion relative to chest width was 2.3–3.1 times greater in QLHA, QMSL and QBSL participants than in LSL participants (p < 0.001). Significant differences between Quechua populations were not observed in either dorsoventral or mediolateral expansions in response to faster f_H_.

### Change in dorsoventral and mediolateral expansions with increasing tidal volume

**Figure 6:**
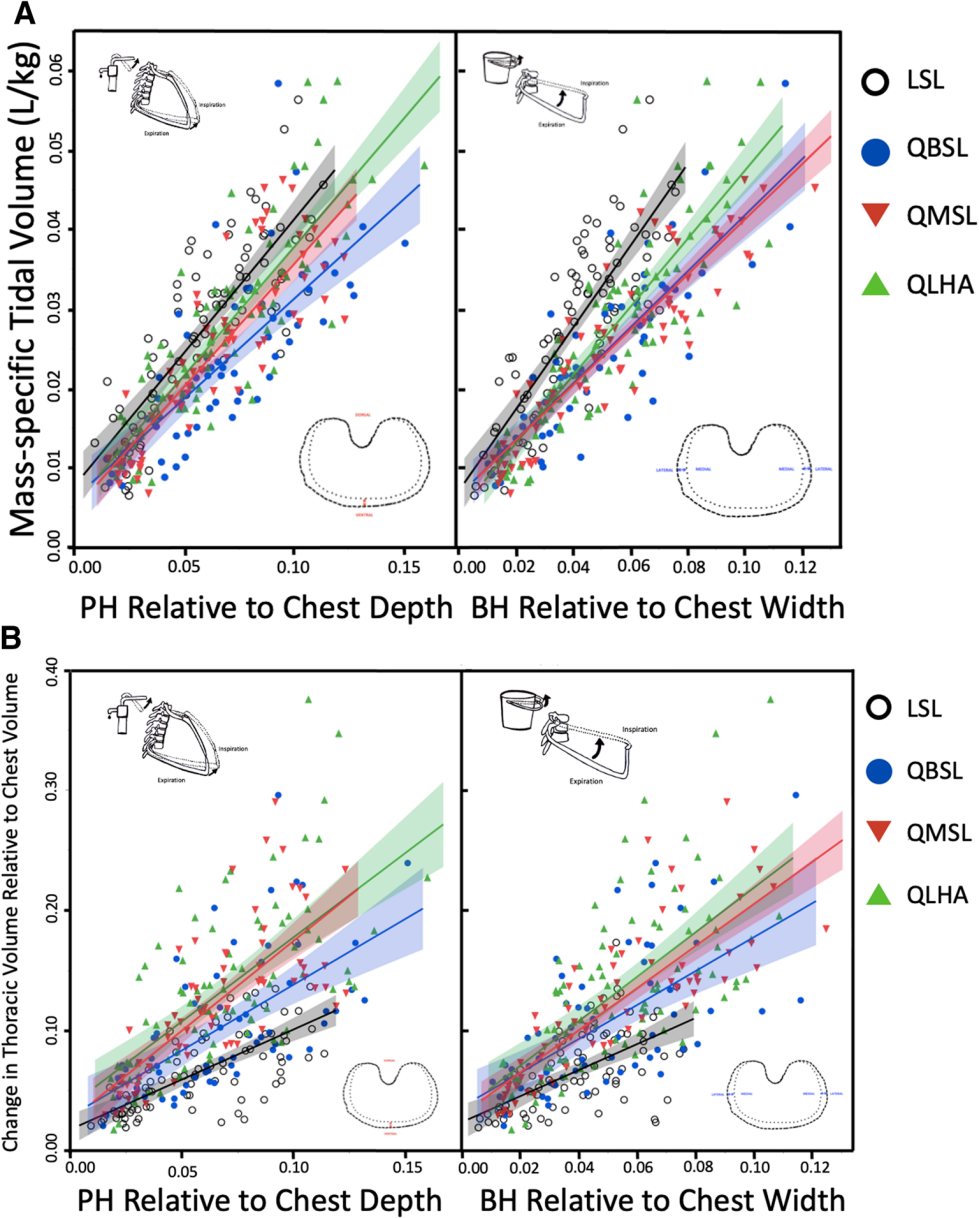
(**A**) Dorsoventral expansion normalized to chest depth (PH) drives increases in thoracic volume. Mass-specific V_T_ (L/kg-BTPS) increases with both dorsoventral and mediolateral thoracic expansion in all populations. Mass-specific V_T_ (L/kg-BTPS) increased with maximum dorsoventral expansion relative to chest depth more in LSL participants (slope ± S. 0.37 ± 0.02, R^2^ = 0.70) than Quechua participants (p < 0.01). QLHA (0.35 ± 0.02, R^2^ = 0.72; p = 0.006) and QMSL (0.32 ± 0.02, R^2^ = 0.73; p = 0.091) increased more than QBSL (0.27 ± 0.02, R^2^ = 0.60). Mass-specific V_T_ (L/kg-BTPS) also increased with maximum mediolateral expansion relative to chest width significantly more in LSL participants (0.51 ± 0.03, R^2^ = 0.71) than QLHA (p = 0.005), QMSL (p < 0.001) and QBSL (p < 0.001) participants. QLHA (0.42 ± 0.02, R^2^ = 0.73) exhibited greater amounts of mediolateral expansion normalized to chest width (BH) relative to QMSL (0.35 ± 0.02, R^2^ = 0.82; p = 0.020) and QBSL (0.36 ± 0.02, R^2^ = 0.73; p = 0.081). QMSL and QBSL did not differ significantly (p = 0.739). Shading represents 95% CLs. (**B**) Change in thoracic volume relative to chest volume increased with maximum dorsoventral expansion relative to chest depth (PH) more in QLHA (p < 0.001), QMSL (p < 0.001) and QBSL (p = 0.101) participants than in LSL participants (slope ± S. 1.03 ± 0.07, R^2^ = 0.47). QLHA (1.64 ± 0.12, R^2^ = 0.44) and QMSL (1.62 ± 0.12, R^2^ = 0.56) exhibited similar amounts of thoracic volume change relative to dorsoventral expansion (p = 0.927), while QBSL (1.27 ± 0.13, R^2^ = 0.37) had less thoracic volume change resulting from similar amounts of dorsoventral expansion than QLHA (p = 0.038) and QMSL (p = 0.042) participants. Change in thoracic volume relative to chest volume increased with maximum mediolateral expansion relative to chest width (BH) more in QLHA (p < 0.001), QMSL (p = 0.015) and QBSL (p = 0.187) participants than in LSL participants (1.35 ± 0.12, R^2^ = 0.34). QLHA (2.07 ± 0.13, R^2^ = 0.43), QMSL (1.76 ± 0.11, R^2^ = 0.68; p = 0.075) and QBSL (1.61 ± 0.15, R^2^ = 0.38; p = 0.024) exhibited similar amounts of thoracic volume change relative to dorsoventral expansion. QMSL and QBSL were not significantly different (p = 0.439). Shading represents 95% CIs. Multiple measures from individual participants are presented together for graphical clarity. Repeated measures in participants are accounted for and fixed effects assessed via non-linear mixed effects models using generalized least squares (see “Statistical analysis”).

Dorsoventral and mediolateral thoracic expansion increased with mass-specific V_T_ (L/kg-BTPS) in all populations (Fig. 6A). Dorsoventral expansion increased approximately 1.1–1.4 times more in LSL participants than in Quechua participants as mass-specific V_T_ increased (p < 0.01), meaning increasing tidal volume required greater amounts of relative dorsoventral expansion in LSL than in Quechua participants. Mediolateral expansion increased approximately 1.2–1.5 times more in LSL participants than in Quechua participants as mass-specific V_T_ increased (p < 0.01). Again, increasing tidal volume required greater amounts of relative mediolateral expansion in LSL individuals than in Quechua participants.

### Dorsoventral and mediolateral thoracic expansions with increasing thoracic volume change

Change in thoracic volume relative to chest volume increased with dorsoventral expansion and mediolateral expansion in all populations (Fig. 6B). Change in thoracic volume increased with maximum dorsoventral expansion relative to chest depth approximately 1.3–1.6 times more in Quechua participants than in LSL participants, meaning dorsoventral expansion in Quechua participants resulted in larger increases in thoracic volume than in LSL participants. QBSL participants had less overall thoracic volume change resulting from similar amounts of dorsoventral expansion than QLHA (p = 0.038) and QMSL (p = 0.042) participants.

Change in thoracic volume relative to chest volume also increased with maximum mediolateral expansion relative to chest width approximately 1.2–1.6 times more in Quechua participants than in LSL participants. Consequently, mediolateral expansion in Quechua participants resulted in larger changes in thoracic volume than in LSL participants. QBSL participants had less overall thoracic volume change resulting from similar amounts of mediolateral expansion than QLHA (p = 0.024) and QMSL (p = 0.439) participants. Overall, dorsoventral and mediolateral chest expansions increased thoracic volume more in Quechua participants than in sea-level lowlanders.

## Discussion

This study’s primary aim was to test if increased oxygen demand during sustained PA selected for increased thoracic ventilation in humans from hypoxic high-altitude environments. Although high-altitude adapted humans are known to have larger lung volumes and physiological adaptations to increase oxygen transport in the body, it is not known if individuals in these populations are able to increase the amount of overall thoracic expansion and contraction per breath as V_T_ increases. To address this question, we took advantage of a natural experiment by comparing rib kinematics during aerobic activity in Quechua individuals, a population generally adapted to high altitude that now includes individuals who live at different elevations and, hence, in different oxygen environments. We found that Quechua participants, regardless of whether they live at high or low altitude, increased thoracic volume change approximately twofold more than low-altitude native participants from the sea-level Boston area (LSL) during moderate-intensity endurance activity despite having absolutely smaller chests (Table 1), thereby supporting our Population-Level Adaptation Hypothesis (Figs. 3, 4A). Greater thoracic volume change was observed in Quechua relative to LSL participants even at rest (Fig. S6), and thoracic ventilation was also found to be greater in Quechua than in LSL participants (Fig. 4B,C).

When controlling for differences in available oxygen in different altitude environments, differences in thoracic function between Quechua populations emerged (Fig. 5). QLHA were able to achieve similar oxygen intake at high altitude as sea-level populations despite every breath of air containing approximately 60% of the oxygen molecules relative to sea level. QLHA achieved this, in part, by increasing thoracic expansion and contraction during each ventilatory cycle (Figs. 3, 4, 5). Quechua living at sea level (QMSL and QBSL participants) were also found to have greater amounts of thoracic volume change relative to oxygen intake compared to LSL participants (Fig. 5). Observed differences in normalized thoracic ventilation between QLHA and QMSL participants suggest that acclimatization to different oxygen environments results in differences in thoracic ventilatory function, supporting our Adulthood Acclimatization Hypothesis. Individuals living in a hypoxic high-altitude environment expand their chests more to breathe. However, because differences are observed between all Quechua populations and LSL participants, increased thoracic ventilation is partially a population-level adaptation.

Although we found population-level differences in ventilatory capacity, this study also found that Quechua living at sea level (QMSL and QBSL participants) had reduced f_R_ and mass-specific ${\dot{\text{V}}}$_E_ at a given f_H_ relative to QLHA and LSL participants (Fig. S4). This finding suggests that Quechua individuals do not need to dramatically increase f_R_ and mass-specific ${\dot{\text{V}}}$_E_ at sea level due to physiological adaptations for increased extraction of oxygen from inhaled air, such as greater hemoglobin-oxygen affinity. Lowlanders at sea level, however, must increase ${\dot{\text{V}}}$_E_ during aerobic activity to a greater extent than Quechua likely because they lack underlying physiological adaptations to increase the efficiency of oxygen diffusion from the lungs into the bloodstream, as also suggested by numerous studies reporting enlarged lung volumes in Quechua populations^22,25,26,32,37,43^.

Regardless of physiological adaptations for oxygen diffusion, Quechua individuals at high altitude and sea level augmented ${\dot{\text{V}}}$_E_ not only through increased diaphragmatic ventilation but also through increased thoracic expansion and contraction. QLHA individuals increased normalized thoracic ventilation the most, followed by QMSL and QBSL individuals, respectively (Fig. 4B). All three Quechua populations increased normalized thoracic ventilation to a greater extent than LSL participants during aerobic activity. We therefore conclude that the thorax contributes to increased ${\dot{\text{V}}}$_E_ more in high-altitude adapted Quechua, regardless of oxygen environment, than in lowlanders, suggesting underlying population-level selection.

In terms of the mechanical bases for differences in these ventilatory capacities, we found that Quechua and lowlander thoraxes functioned differently both in terms of dorsoventral and mediolateral expansion, as predicted based on the shape of the chest. Andean populations have been found to have both wider and deeper chests^28,31–36^, and the Quechua individuals included in this study were no exception (Table 1 and Table S1). We found that Quechua participants who had grown up or lived at high altitude had deeper chests than LSL participants. Quechua populations generally had both deeper and wider chests than LSL participants despite being shorter and having less body mass, demonstrating population-level inheritable thoracic adaptations to high altitude. Correspondingly, relative mediolateral and dorsoventral chest expansions were greater in all Quechua than in LSL participants (Fig. 6). However, we also found that QLHA and QMSL participants had deeper chests than QBSL participants, an additional developmental effect on chest shape. The functional consequence of deeper chests in Quechua was increased dorsoventral expansion during ventilation in Quechua populations that had grown up at high altitude, supporting the Developmental Adaptation Hypothesis (Fig. 6). Overall, QLHA and QMSL participants had greater observed dorsoventral thoracic expansion than QBSL participants, though similar amounts of mediolateral expansion.

This study has several limitations. First, thoracic expansion and contraction was quantified using wearable devices made from polymer nanocomposites that measure change in thoracic circumference at three different points on the chest (Fig. 1B,C). Overall thoracic volume was calculated through time using the devices^44,45^ and by modeling the thorax as a multi-part, truncated cone (adapted from Ruff^46^; Fig. S10). This method was shown to be reliably accurate (Fig. S8) compared to validated 3D motion capture methods^4^. However, we estimated thoracic expansion using three separate models to estimate maximum and minimum possible measurements of thoracic expansion (Fig. S8). The results presented here reflect the findings of a more conservative model (Model 2), meaning thoracic expansion in our Quechua participants could be even greater than presented. Regardless, even the most conservative estimates of thoracic expansion and contraction during ventilation obtained using the nanocomposite devices suggest significant differences between high-altitude adapted Quechua populations and LSL participants, in agreement with significant differences in PH and BH motion observed between the populations. Hence, we are confident that differences in thoracic function exist between high-altitude and sea-level populations. Second, the use of surface devices can lead to error in kinematic measurements due to underlying subcutaneous fat and muscle. However, because we measured change in volume rather than volume itself, our results were likely unaffected by these issues. Third, measurements of thoracic expansion and contraction were obtained only during sustained walking and running on a treadmill. Running speed and heart rate were constrained to ensure Quechua participant safety, especially for participants who were unfamiliar with running on a treadmill. However, moderate-intensity aerobic activities such as carrying heavy loads and trekking up inclines were not measured. That said, our study does measure ventilation in response to increasing heart rate rather than relying on measurements taken during specific gaits, making deviations in thoracic function during other moderate-intensity endurance activities unlikely. Fourth, Quechua ancestry was not directly measured. However, there is little reason to doubt Quechua ancestry in our sample populations as the inclusion criteria for participation in the study were thorough, including a family history that determined Quechua ancestry through established methods such as knowledge of the Quechua language and surname analysis^27,38,47^. Most importantly, we compared thoracic function only between male Quechua participants and lowland males living within the greater Boston area. Although our research questions concerning thoracic function apply equally to women, we were not able to measure women in this study because to measure thorax movements accurately nanopolymer devices need to be applied to the thorax of shirtless participants. Future research using alternative methods are needed to test for differences in thoracic expansion between males and females. Additionally, it would also be useful to study other populations with long-term exposure to high altitude, such as Tibetans. Finally, low-altitude short-term visitors to high altitude and low-altitude populations living and acclimatized to high-altitude environments were not studied. Expanding future research to include more varied populations will help us better understand the plasticity of thoracic function in response to different oxygen environments.

Our measured differences in thoracic biomechanics between human populations with different adaptations to altitude and living in different oxygen environments have broader implications for the effects of oxygen demands on human evolution. Evidence of derived concavo-convex costovertebral joints in *Homo erectus*, *Homo neanderthalensis* and *Homo sapiens* suggests that the genus *Homo* evolved to supplement diaphragmatic ventilation with thoracic ventilation, which was likely constrained in australopiths and other previous hominin ancestors^4^. Specifically, while all mammals use diaphragmatic breathing to increase V_T_ with increasing oxygen demand, bipedal humans expand the thorax using both dorsoventral and mediolateral rib motions. Our study of high-altitude and low-altitude populations demonstrates that a combination of acclimatization to novel oxygen environments, developmental adaptation, and population-level adaptation influence thoracic function in response to increased oxygen demand. Changes in chest function that increase thoracic ventilation, the same modifications evident in all humans relative to apes^4^, are more pronounced in humans living at high altitude, suggesting that the human thorax has an impressive degree of phenotypic and functional plasticity. As a species, humans have evolved to use the thorax to ventilate while performing sustained moderate to high-intensity aerobic activities like running. Thus, it is hardly surprising that thoracic adaptations for ventilation, much like other respiratory and physiological modifications, are enhanced in a population living in an extreme hypoxic environment. Selection for endurance activity apparently changed how we breathe and allowed human life to spread from the savannah to top of the world.

## Online methods

### Participants

Healthy, adult males (age 19–30 years old) with no history of major neuromuscular, cardiovascular or respiratory disease were recruited from three different Quechua participant populations and compared to sea-level lowland participants (Fig. 1, Table 1 and Table S1). Although this study’s research questions concerning thoracic function apply equally to women and men, we were not able to measure women because rib motion can be measured only in shirtless participants. All participants provided written informed consent, and prior approval for the experiments was obtained from the Committees on the Use of Human Subjects at Harvard University, Cambridge, Massachusetts, and Universidad Peruana Cayetano Heredia, Lima, Peru. All research was performed in accordance with relevant guidelines and regulations. Recruited participants were informed of the study by word-of-mouth through recruiters who were members of the local community. Two such recruiters, one in Lima, Peru, and one in Cerro de Pasco, Peru, were responsible for enrolling participants, the majority of whom were either students or university workers. Both recruiters had been involved in previous research projects conducted by the Universidad Peruana Cayetano Heredia. Potential participants were screened via a brief clinical history and physical examination for conditions contraindicating participation in the study protocols. Inclusion criteria were being 18–30 years old, male, fluent in Spanish, and having Quechua ancestry. Exclusion criteria included any injury or pain that impaired their gait or stability, dizziness, fever, or discomfort with walking on a treadmill. We also excluded participants who had been told by their physician to limit physical activity or who had reportedly passed out during exercise during the last 3 years. No minimum level of regular exercise was required to enroll in the study, but individuals with previously diagnosed heart conditions, exertional chest pain, high blood pressure, or using medications to treat high blood pressure were excluded. No professional athletes or individuals with significant athletic training during their lifetime enrolled in the Peruvian or Boston portions of the study. Inclusion criteria were not specifically revealed to participants during the participant interview to better establish an accurate family and migration history. Participants were given a questionnaire to determine the altitude environments at which they had lived for prolonged periods of time and to establish family history. All Quechua individuals included in this study satisfied at least two of three criteria to establish Quechua ancestry^38^: (1) presence of at least one or more Quechua surnames from both parental lineages; (2) participants identified themselves as having ancestors from the highlands; and, (3) knowledge of the Quechua language by themselves and/or at least one of their parents or grandparents (Quechua is not taught at school and it is only learned via exposure to the language through family members).

Three Peruvian populations were studied and compared to a previously studied lowlander population from Boston^4^. Population 1 consisted of Quechua participants who were born and raised at sea level (QBSL; n = 16). QBSL participants accepted into the study met the following criteria: (1) they were born in Lima or near sea level, (2) both parents were born at altitude greater than 3000 m, (3) both sets of grandparents were born at altitude greater than 3000 m, and (4) at least 95% of their growth and development period (birth to 18 years) was spent in Lima or at sea level. Highland visits by these participants were limited to no more than 2 weeks/year total. If any participant spent more than two uninterrupted months in the highlands at any time during growth and development, he was excluded from the study.

Population 2 consisted of Quechua participants who were born and raised above 3000 m but who migrated permanently to sea level (QMSL; n = 17). Participant recruitment and interview strategies were the same as those employed with QBSL participants. QMSL participants accepted into the study met the following criteria: (1) they were born and raised at altitude greater than 3000 m; (2) both parents were born at altitude greater than 3000 m; (3) both sets of grandparents were born at altitude greater than 3000 m; (4) at least 83% of their growth and development period was spent at altitude greater than 3000 m; and, (5) had lived at sea level for at least 4 years.

Population 3 consisted of Quechua participants born above 3000 m living at high altitude (QLHA; n = 20). Participant recruitment and interview strategies were the same as those employed with QBSL and QMSL participants. QLHA participants accepted into the study met the following criteria: (1) they were born and raised at altitude greater than 3000 m; (2) both parents were born at altitude greater than 3000 m; (3) both sets of grandparents were born at altitude greater than 3000 m; and (4) at least 95% of their growth and development period was spent at altitude greater than 3000 m. None of these participants was involved in lead mining activities, reducing the potential damaging effects on the respiratory system inherent with the occupation and which have affected previous research conducted in Cerro de Pasco, and no participants had any symptoms of Chronic Mountain Sickness (CMS).

### Anthropometry

We measured height, body mass, chest width, chest circumference, and chest depth in all participants^48^. Chest width, chest circumference, and chest depth were measured at three levels on the thorax in Quechua participants. Forced vital capacity (FVC) and forced expiratory volume (FEV_1_) were also measured.

### Kinematics/experimental trials

Kinematic data was collected in two separate experiments. Experiment 1 was conducted in Lima, Peru (elev. 154 m), with Quechua participants who were born and raised above 3000 m but who migrated permanently to sea level (QMSL; n = 17) and Quechua participants who were born and raised at sea level (QBSL; n = 16). Experiment 2 was conducted at Cerro de Pasco, Peru (elev. 4340 m) with Quechua participants born above 3000 m living at high altitude (QLHA; n = 20).

Participant ${\dot{\text{V}}}$O_2_ and associated heart rates (f_H_) were measured using a portable respirometry system (Sable Systems, North Las Vegas, NV, USA) and f_H_ monitor (Suunto, Vantaa, Finland). As part of a method for estimating f_H,MAX_, participants carried out a 1-mile, steady-state jog while wearing the devices. Running speed was restricted to slower than 8.0 min/mile to prevent possible injury, to familiarize participants with walking and running on a treadmill, and to correspond with ${\dot{\text{V}}}$O_2_ estimation protocols^49^. ${\dot{\text{V}}}$O_2MAX_ was estimated based on test performance, sex and body metrics using an accepted validation model^49^. Predicted maximal f_H_ was determined based on calculated ${\dot{\text{V}}}$O_2MAX_ and the ${\dot{\text{V}}}$O_2_ and associated f_H_ measured during the test ($f_{H,MAX} = \frac{{V0_{2MAX,estimated} }}{{VO_{2,test} }} \times f_{H,test}$). Specific f_H_ targets for the remainder of the study were calculated from this maximal f_H_ to allow for a broad range of aerobic intensities to be measured corresponding with percentages of ${\dot{\text{V}}}$O_2MAX._

All participants were outfitted with newly developed, wearable devices made from polymer nanocomposites (portable C-Stretch strain sensors; Bando Chemical Industries, Ltd., Japan) that measure thoracic expansion and contraction. The nanocomposite devices were used to measure change in thoracic circumference at different points on the chest, as well as independent dorsoventral and mediolateral thoracic expansions (Fig. S9). The overall thoracic volume was calculated through time using the devices^44,45^. Using a multi-part, truncated cone model to determine resting volume of the thorax (adapted from Ruff^46^; Fig. S10), the nanocomposite devices accurately and reliably measure changes in thoracic volume (orthogonal fit ratio = 0.997; see “Statistical analysis”, below).

All participants completed a series of randomized trials on a portable treadmill at rest and approximately 40%, 60% and 80% of estimated maximum f_H_ with treadmill speed adjusted based on real-time f_H_ measurements. Participants walked or ran for 5–10 min per trial. During each experimental condition, measurements of inspiratory flow and volume (tidal volume; V_T_) were taken using a spirometer (ML311, ADInstruments, Colorado Springs, CO, USA) attached to a one-way flow respirometry mask and using Lab Chart spirometry software (AD Instruments). Resting respiratory frequency, resting V_T_, maximum voluntary inspiratory volume, and maximum voluntary expiratory volume were measured using the spirometry apparatus attached to a two-way flow mouthpiece.

### Statistical analysis

To assess the reliability and accuracy of our volumetric calculations of thoracic volume using the nanocomposite devices, we used the above methods to measure the volume in human participants (n = 5) in a lab. Our field volumetric assessment method was then tested using an orthogonal regression test examining the linear relationship between two continuous variables and comparing expected volume measurements, measured using validated 3D motion capture methods^4^, with those observed/measured using the nanocomposite devices.

Data analysis was performed in JMP Pro 15 (SAS Institute Inc., Cary, NC, USA) and R^50^. Because mammalian lung volume scales isometrically with body mass^42^, V_T_ was standardized by body mass to allow for comparisons across participants and across populations. Likewise, maximum dorsoventral and mediolateral thoracic expansion was standardized by chest depth and width, respectively. Changes in thoracic volume were measured as the difference in thoracic volume between inspiration and expiration. The change in thoracic volume per breath was then standardized to calculated volume of the chest based on the multi-part, truncated cone model (Fig. S10) and calculated as the percentage change to allow comparisons across populations.

To account for repeated measures and non-parametric data, non-linear mixed effects models using generalized least squares were used to assess fixed effects, including age, f_H_ and population, on thoracic response variables across populations. Individual subject ID was included as a random effect and subjects were treated as a random sample from their larger populations. Differences in slope between groups were assessed using a repeated measures ANCOVA. Differences in resting thoracic volume change were assessed using a pairwise Wilcoxon test.

## Supplementary Information

Supplementary Information.

## Data Availability

Data is available through the Dryad digital repository.

## References

[1] Bramble DM, Lieberman DE (2004). Endurance running and the evolution of Homo. Nature.

[2] Lieberman DE (2015). Human locomotion and heat loss: An evolutionary perspective. Comp. Physiol..

[3] Shave RE (2019). Selection of endurance capabilities and the trade-off between pressure and volume in the human heart. Proc. Natl. Acad. Sci..

[4] Callison WE, Holowka NB, Lieberman DE (2019). Thoracic adaptations for ventilation in humans and other cursorial mammals. J. Exp. Biol..

[5] Bulbulian R, Wilcox A, Darabos B (1986). Anaerobic contribution to distance running performance of trained cross-country athletes. Med. Sci. Sport Exerc..

[6] Weibel ER (1984). The Pathway for Oxygen.

[7] Berry MJ, Dunn CJ, Pittman CL, Kerr WC, Adair NE (1996). Increased ventilation in runners during running as compared to walking at similar metabolic rates. Eur. J. Appl. Physiol..

[8] Brutsaert TD (2019). Association of EGLN1 gene with high aerobic capacity in Peruvian Quechua at high altitude. Proc. Natl. Acad. Sci..

[9] Moseley ME (2001). The Incas and their Ancestors.

[10] Hartley LH, Alexander JK, Modelski M, Grover RF (1967). Subnormal cardiac output at rest and during exercise in residents at 3,100 meters altitude. J. Appl. Physiol..

[11] Moret P, Covarrubias E, Coudert J, Duchosal F (1972). Cardiocirculation adaptation to chronic hypoxia III. Comparative study of cardiac output, pulmonary and systematic circulation between sea level and high altitude residents. Acta Cardiol..

[12] Naeije R (2010). Physiological adaptation of the cardiovascular system to high altitude. Prog. Cardiovasc. Dis..

[13] Abbrecht PH, Littell JK (1972). Plasma erythropoietin in men and mice during acclimatization to different altitudes. J. Appl. Physiol..

[14] Beall CM (2001). Adaptation to altitude: A current assessment. Annu. Rev. Anthropol..

[15] Beall CM (2007). Two routes to functional adaptation: Tibetan and Andean high-altitude natives. Proc. Natl. Acad. Sci..

[16] Frisancho AR (1995). Developmental, genetic, and environmental components of aerobic capacity at high altitude. Am. J. Phys. Anthropol..

[17] Hoppeler H, Vogt M, Weibel ER, Fluck M (2003). Response of skeletal muscle mitochondria to hypoxia. Exp. Physiol..

[18] Pugh LGCE (1964). Blood volume and hemoglobin concentration at altitudes above 18,000 ft (5,500 m). J. Physiol..

[19] Spievogel H, Otero-Calderon L, Calderon G, Hartmann R, Cudkowicz L (1969). The effects of high altitude on pulmonary hypertension of cardiopathies, at La Paz, Bolivia. Respiration.

[20] Ward MP, Milledge JS, West JB (2000). High Altitude Medicine and Physiology.

[21] Boyce AJ, Haight JSJ, Rimmer DB, Harrison GA (1974). Respiratory function in Peruvian Quechua Indians. Ann. Hum. Biol..

[22] Hurtado A, Dill DB, Adolph EF, Wilber CG (1964). Handbook of Physiology: Adaptation to Environment.

[23] Brody J, Lahiri S, Simpser M, Motoyama EK, Valasquez T (1977). Lung elasticity and airway dynamics in Peruvian natives to high altitude. J. Appl. Physiol..

[24] Cruz J (1973). Mechanics of breathing in high altitude and sea level subjects. Respir. Physiol..

[25] Frisancho AR (1969). Human growth and pulmonary function of a high altitude Peruvian Quechua population. Hum. Biol..

[26] Frisancho AR (1997). Developmental, genetic and environmental components of lung volumes at high altitude. Am. J. Hum. Biol..

[27] Greksa LP (1996). Evidence for a genetic basis to the enhanced total lung capacity of Andean highlanders. Hum. Biol..

[28] Greksa LP, Spielvogel H, Caceres E (1994). Total lung capacity in young highlanders of Aymara ancestry. Am. J. Phys. Anthropol..

[29] Kiyamu M (2012). Developmental and genetic components explain enhanced pulmonary volumes of female Peruvian Quechua. Am. J. Phys. Anthropol..

[30] Lahiri S (1976). Relative role of environmental and genetic factors in respiratory adaptation to high altitude. Nature.

[31] Greksa LP (1986). Chest morphology of young Bolivian high-altitude residents of European ancestry. Hum. Biol..

[32] Mueller WH, Schull VN, Schull WJ, Soto P, Rothhammer F (1978). A multinational Andean Genetic and Health Program: Growth and development in an hypoxic environment. Ann. Hum. Biol..

[33] Niermeyer S, Zamudio S, Moore LG, Hornbein T, Schoene R (2001). High Altitude: An Exploration of Human Adaptation.

[34] Palomino H, Mueller WH, Schull WJ (1979). Altitude, heredity and body proportions in northern Chile. Am. J. Phys. Anthropol..

[35] Pyzuk M, Turusbekow BT, Brjancewa LA (1967). Certain properties of the respiratory system in school children in various altitude and climatic conditions. Hum. Biol..

[36] Stinson S (1985). Chest dimensions of European and Aymara children at high altitude. Ann. Hum. Biol..

[37] Brutsaert TD (2004). Effects of birthplace and individual genetic admixture on lung volume and exercise phenotypes of Peruvian Quechua. Am. J. Phys. Anthropol..

[38] Kiyamu M, Elías G, Leon-Velarde F, Rivera-Chira M, Brutsaert TD (2015). Aerobic capacity of Peruvian Quechua: A test of the developmental adaptation hypothesis. Am. J. Phys. Anthropol..

[39] Harrison GA, Baker PT, Weiner JS (1966). The Biology of Human Adaptability.

[40] Bateson P, Gluckman P, Hanson M (2014). The biology of developmental plasticity and the Predictive Adaptive Response hypothesis. J. Physiol..

[41] Mazess RB, Watts ES, Johnston FE, Lasker GW (1975). Biosocial Interrelation in Population Adaptation.

[42] Gehr P (1981). Design of the mammalian respiratory system. V. Scaling morphometric pulmonary diffusing capacity to body mass: Wild and domestic mammals. Respir. Physiol..

[43] Hurtado A (1932). Respiratory adaptation in the Indian natives of the Peruvian Andes: Studies at high altitude. Am. J. Phys. Anthropol..

[44] Banzett R, Mahan S, Garner D, Brughera A, Loring S (1995). A simple and reliable method to calibrate respiratory magnetometers and Respitrace. J. Appl. Physiol..

[45] Binks A, Banzett R, Duvivier C (2007). An inexpensive, MRI compatible device to measure tidal volume from chest-wall circumference. Physiol. Meas..

[46] Ruff C (1991). Climate and body shape in hominid evolution. J. Hum. Evol..

[47] Chakraborty R, Barton SA, Ferrell RE, Schull WJ (1989). Ethnicity determination by names among the Aymara of Chile and Bolivia. Hum. Biol..

[48] Tanner JM, Hiernaux J, Jarman S, Weiner JS (1969). Human Biology: A Guide to Field Methods.

[49] George J, Vehrs PR, Allsen PE, Fellingham GW, Fischer AG (1993). VO2MAX estimation from a submaximal 1-mile track jog for fit college-age individuals. Med. Sci. Sport Exerc..

[50] Team, R. C. *R: A language and environment for statistical computing* (2014).

