# Supplementary material for: Comparing high versus low-altitude populations to test human adaptations for increased ventilation during sustained aerobic activity
Source: Sci Rep. 2022 Jul 1;12:11148. doi: 10.1038/s41598-022-13263-5 (PMC9249887; doi:10.1038/s41598-022-13263-5)
Supplement: Supplementary file 1 — Supplementary Information. [file 41598_2022_13263_MOESM1_ESM.docx]

**Supplementary Information**

for

**Comparing high versus low-altitude populations to test human adaptations for increased ventilation during sustained aerobic activity.**

**Authors:** W. Éamon Callison, Melisa Kiyamu, Francisco C. Villafuerte, Tom D. Brutsaert, and Daniel E. Lieberman*

*corresponding author

**Biomechanical Model: Thoracic Volume Change Due to Movement at the Costovertebral Joint**

To test how thoracic morphology might affect changes in thoracic volume and thereby affect the function of the thorax, a quantitative biomechanical model can be used to characterizes how movements of the ribs at the costovertebral joints of the spine, coupled with the dorsoventral and mediolateral dimensions of the ribs, yield predictable and measurable thoracic expansions in the sagittal (pump-handle) and coronal (bucket-handle) planes (Fig. S1). Using Ruff’s model of the body as a cylinder (Ruff 1991), changes in thoracic volume that occur from a pair of ribs’ motions can be calculated as a function of the change in area between corresponding ribs (ΔA) and a change in height of the rib pair (Δh) (Fig. S1):

ΔV_Thorax_ = (ΔA)(Δh) (**Eq. S1**)

During inspiration, the rib rotates around its neck axis at the rib’s origin within the costo-vertebral joint (Jordanoglou 1970). The distal edge of the rib creates an arc (P_O_P_f_ in lateral view, Q_O_Q_f_ in dorsal view) in the plane of rotation (Fig. S1). The chord of this arc is a spatial vector (S in lateral view, M in dorsal view) representing the change in position of the distal end of the rib during inspiration (Jordanoglou 1970). Rotation of the rib by a certain degree (θ in lateral view, Φ in dorsal view) results in a calculable change in distance between the initial position and final positions of the distal end of the rib (represented by P_O_ and P_f_ in lateral view, Q_O_ and Q_f_ in dorsal view; Fig. S1). The lines bisecting angle θ and angle Φ intersect chord S and chord M at right angles and also result in angle ι and angle Ψ with respect to the body midline. Consequently, the change in the height of the rib (Δh) is:

Δh = S sin(ι+θ/2) (**Eq. S2**)

The change in the anteroposterior length of the thorax (Δy) is:

Δy = S cos(ι+θ/2) (**Eq. S3**)

The change in the lateral width of the thorax (Δx) is:

Δx = 2M cos(Ψ+Φ/2) (**Eq. S4**)

By modeling the thorax as an ellipse on the transverse plane, the change in area contained within a given rib pair is:

ΔA = π (Δx)(Δy) (**Eq. S5**)

ΔA = π (2M cos(Ψ+Φ/2))(S cos(ι+θ/2)) (**Eq. S6**)

From Eq. S1, the volumetric change resulting from the movement of a rib pair is:

ΔV_Thorax_ = [π(2M cos(Ψ+Φ/2))(S cos(ι+θ/2))] [S sin(ι+θ/2)] (**Eq. S7**)

with Φ being the change in the vertical angle of the rib pair, Ψ being the initial vertical angle of the ribs, θ being the change in horizontal angle of the rib pair, ι being the initial horizontal angle of the ribs, M being the vertical secant distance, and S being the horizontal secant distance (Fig. S2). Overall change in thoracic volume is thus the sum of volumetric change from each rib pair during inspiration. The costovertebral joint elements’ increased included angle coupled with closer ratios of the rib articular facets and the vertebral articular facets result in greater rotational ability of the ribs (θ and Φ) and, from Eq. S7, larger possible changes in the volume of the thorax.

An anatomical model of how thoracic volume changes predicts differences in ventilation between species. Anatomical differences that enable greater changes in thoracic volume, though beneficial in enabling aerobically demanding activities such as endurance running, may unavoidably increase work by the muscles that cause these respiratory movements. This anatomical model links joint morphology to rib movement and volumetric change and allows for analysis of fossil rib and vertebral elements in order to predict how thoracic function and ventilation have changed over the course of human evolution.

J. Jordanoglou. Vector analysis of rib movement. *Respiration Physiology*, 10, 109-120 (1970).

C. Ruff. Climate and body shape in hominid evolution. *Journal of Human Evolution,* 21, 81-105 (1991).


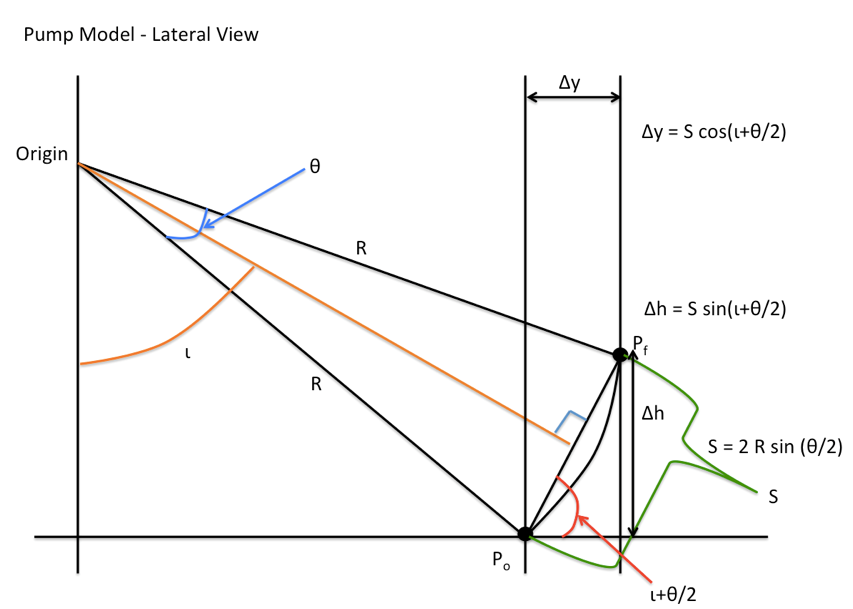

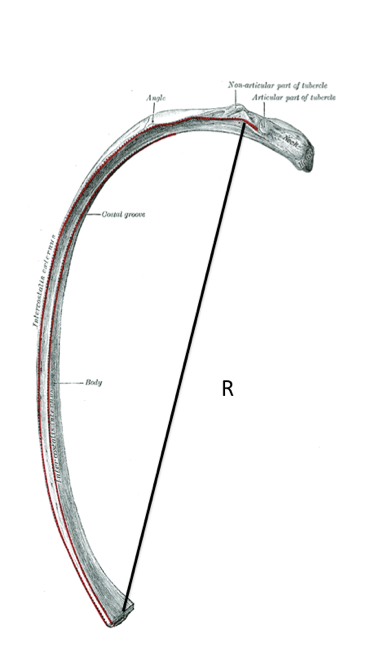
**
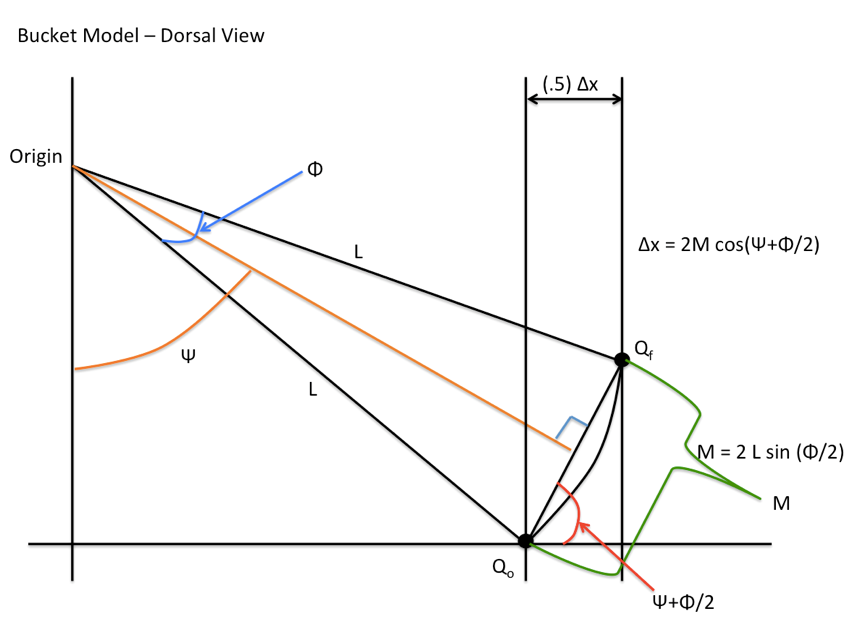

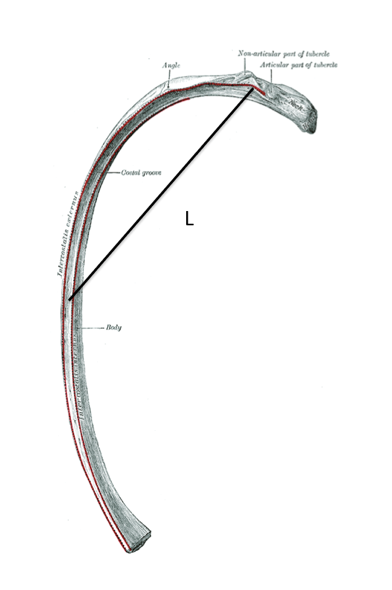
**

**Figure S1:** Joint axis of rotation resulting in pump-handle and bucket-handle rib displacements. During inspiration the rib rotates around its neck axis at the rib’s origin within the costovertebral joint. The change in the height of the rib (Δh) is Δh = S sin(ι+θ/2). The change in the anteroposterior length of the thorax (Δy) is: Δy = S cos(ι+θ/2). The change in the lateral width of the thorax (Δx) is Δx = 2M cos(Ψ+Φ/2).

**
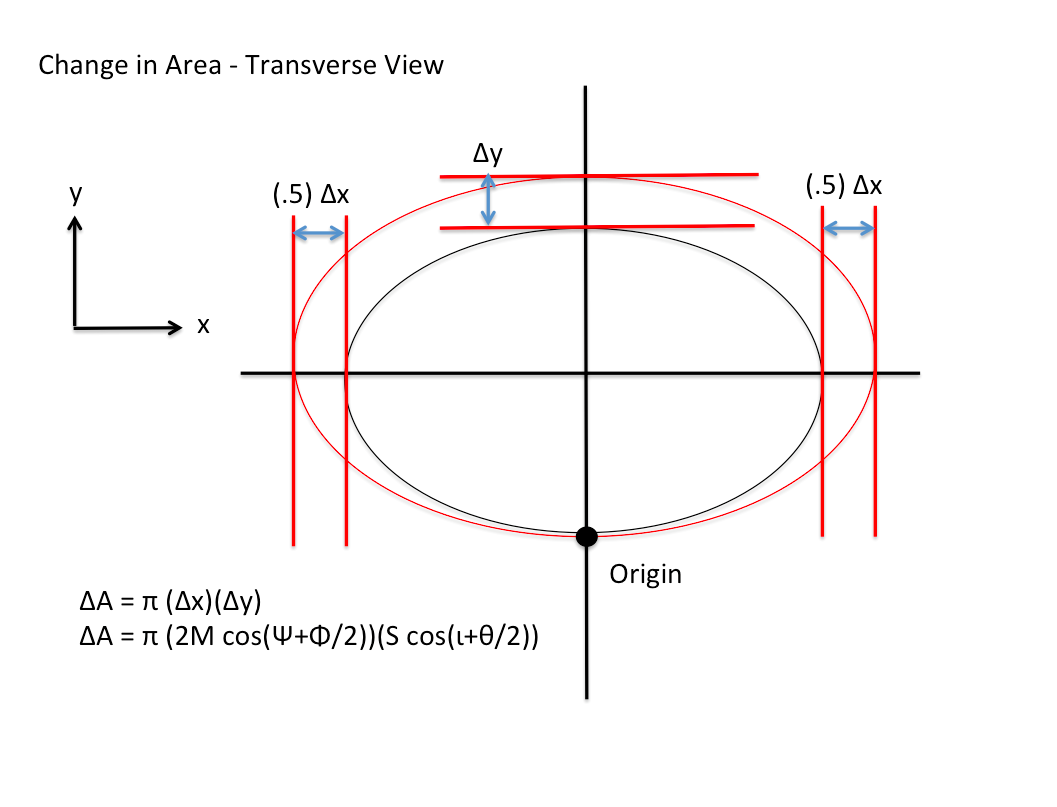
**

**Figure S2:** Transverse thoracic area calculation. By modeling the thorax as an ellipse on the transverse plane, the change in area contained within a given rib pair (ΔA) is ΔA = π (Δx)(Δy) = π (2M cos(Ψ+Φ/2))(S cos(ι+θ/2)).


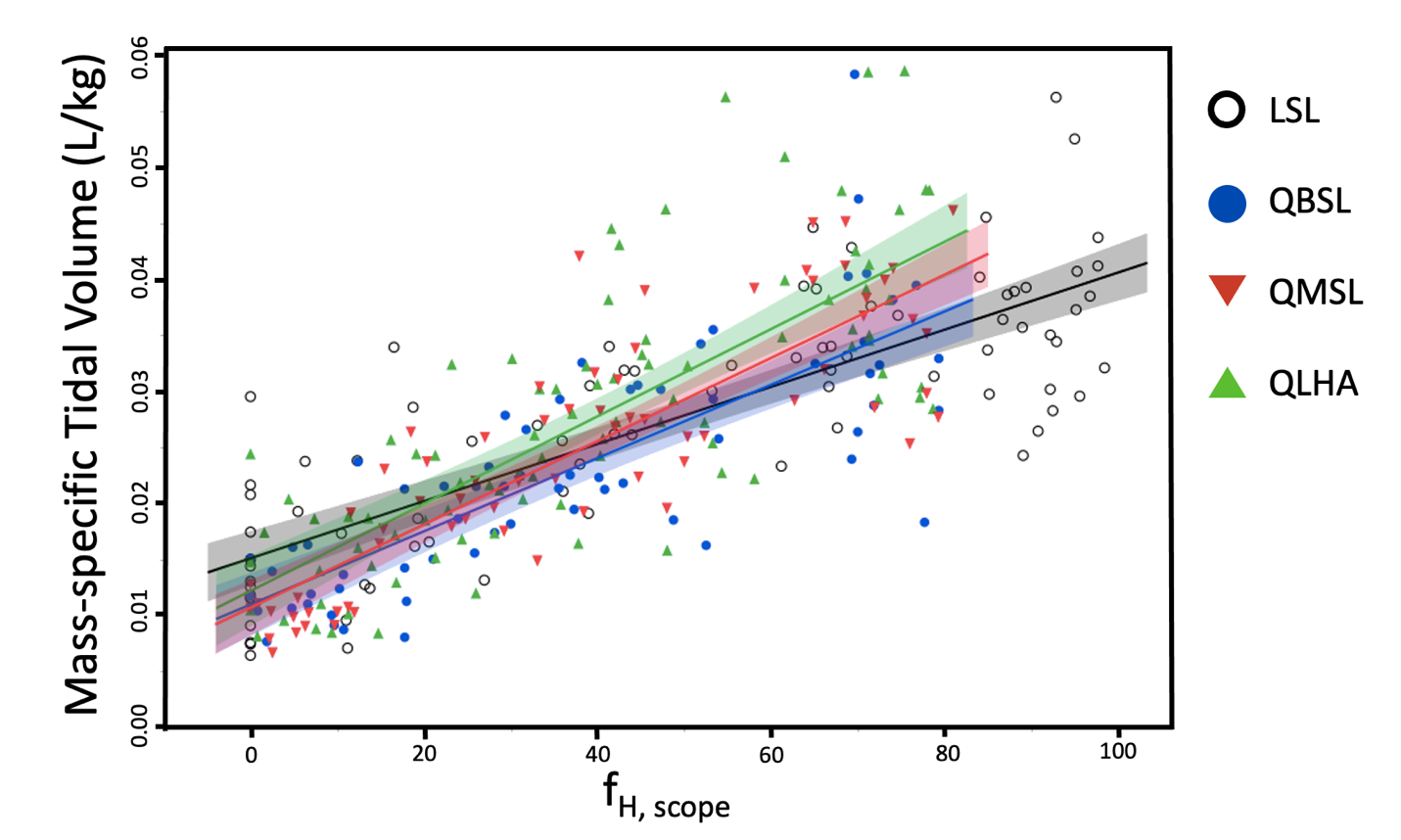


**Figure S3.** Mass-specific V_T_ (L/kg) increases with f_H_ and, hence, V̇O_2_ across populations.

|  | QLHA (n=20) | QMSL (n=17) | QBSL (n=16) | LSL (n=15) |
| --- | --- | --- | --- | --- |
| Age (years) | 23.15 (± 3.23)  **^NS^**^(QMSL),^  **^NS^**^(QBSL),^  **^NS^**^(LSL)^ | 24.29 (± 3.27)  **^NS^**^(QLHA),^  **^NS^**^(QBSL),^  **^NS^**^(LSL)^ | 24.06 (± 3.40)  **^NS^**^(QLHA),^  **^NS^**^(QMSL),^  **^NS^**^(LSL)^ | 22.73 (± 4.01)  **^NS^**^(QLHA),^  **^NS^**^(QMSL),^  **^NS^**^(QBSL)^ |
| Standing Height (cm) | 164.78 (± 5.82)  **^NS^**^(QMSL),^  **^NS^**^(QBSL),^  ***^(LSL)^ | 165.57 (± 5.01)  **^NS^**^(QLHA),^  **^NS^**^(QBSL),^  ***^( LSL)^ | 166.09 (± 5.49)  **^NS^**^(QLHA),^  **^NS^**^(QMSL),^  ***^( LSL)^ | 178.93 (± 5.65)  *******^(QLHA),^  *******^(QMSL),^  *******^(QBSL)^ |
| Body Mass (kg) | 64.94 (± 10.61)  **^NS^**^(QMSL),^  **^NS^**^(QBSL),^  *^( LSL)^ | 67.97 (± 8.10)  **^NS^**^(QLHA),^  **^NS^**^(QBSL),^  **^NS^**^( LSL)^ | 70.83 (± 9.98)  **^NS^**^(QLHA)^**^,^**  **^NS^**^(QMSL)^**^,^**  **^NS^**^(LSL),^ | 72.51 (± 8.51)  *****^(QLHA),^  **^NS^**^(QMSL),^  **^NS^**^(QBSL)^ |
| Hip/Waist Ratio | 1.12 (± 0.06)  **^NS^**^(QMSL),^  **^NS^**^(QBSL)^ | 1.11 (± 0.05)  **^NS^**^(QLHA),^  **^NS^**^(QBSL)^ | 1.12 (± 0.07)  **^NS^**^(QLHA),^  **^NS^**^(QMSL)^ |  |
| Sitting Height (cm) | 88.68 (± 2.82)  **^NS^**^(QMSL),^  **^NS^**^(QBSL),^  ***^( LSL)^ | 88.41 (± 2.16)  **^NS^**^(QLHA),^  **^NS^**^(QBSL),^  ***^( LSL)^ | 89.36 (± 3.55)  **^NS^**^(QLHA),^  **^NS^**^(QMSL),^  **^(LSL)^ | 94.12 (± 2.97)  *******^(QLHA),^  *******^(QMSL),^  ******^(QBSL)^ |
| BMI | 23.89 (± 3.47)  **^NS^**^(QMSL),^  **^NS^**^(QBSL),^  **^NS^**^(LSL)^ | 24.79 (± 2.72)  **^NS^**^(QLHA),^  **^NS^**^(QBSL),^  *****^(LSL)^ | 25.67 (± 3.22)  **^NS^**^(QLHA),^  **^NS^**^(QMSL),^  *****^(LSL)^ | 22.70 (± 3.04)  **^NS^**^(QLHA),^  *****^(QMSL),^  *****^(QBSL)^ |
| Chest depth (cm) | 24.91 (± 4.10)  **^NS^**^(QMSL),^  **^NS^**^(QBSL),^  **^NS^**^(LSL)^ | 25.56 (± 1.64)  **^NS^**^(QLHA),^  *****^(QBSL),^  *****^(LSL)^ | 23.84 (± 2.83)  **^NS^**^(QLHA),^  *^(QMSL),^  **^NS^**^(LSL)^ | 23.89 (± 1.72)  **^NS^**^(QLHA),^  *****^(QMSL),^  **^NS^**^(QBSL)^ |
| Upper chest width (cm) | 32.97 (± 1.40)  **^NS^**^(QMSL),^  **^NS^**^(QBSL)^ | 33.88 (± 2.92)  **^NS^**^(QLHA),^  **^NS^**^(QBSL)^ | 33.71 (± 2.79)  **^NS^**^(QLHA)^**^,^**  **^NS^**^(QMSL)^ |  |
| Middle chest width (cm) | 31.20 (± 1.55)  **^NS^**^(QMSL),^  **^NS^**^(QBSL),^  **^NS^**^(LSL)^ | 31.00 (± 3.18)  **^NS^**^(QLHA),^  **^NS^**^(QBSL),^  **^NS^**^(LSL)^ | 32.14 (± 2.79)  **^NS^**^(QLHA),^  **^NS^**^(QMSL),^  **^NS^**^(LSL)^ | 30.87 (± 2.04)  **^NS^**^(QLHA),^  **^NS^**^(QMSL),^  **^NS^**^(QBSL)^ |
| Lower chest width (cm) | 30.33 (± 2.63)  **^NS^**^(QMSL),^  **^NS^**^(QBSL)^ | 29.25 (± 3.42)  **^NS^**^(QLHA),^  **^NS^**^(QBSL)^ | 30.66 (± 2.70)  **^NS^**^(QLHA),^  **^NS^**^(QMSL)^ |  |
| Upper chest circumference (cm) | 93.73 (± 5.57)  *****^(QMSL),^  **^NS^**^(QBSL)^ | 98.39 (± 5.51)  *****^(QLHA),^  **^NS^**^(QBSL)^ | 97.55 (± 6.19)  **^NS^**^(QBSL),^  **^NS^**^(QMSL)^ |  |
| Middle chest circumference (cm) | 88.39 (± 5.75)  **^NS^**^(QMSL),^  **^NS^**^(QBSL)^ | 87.30 (± 15.06)  **^NS^**^(QLHA),^  **^NS^**^(QBSL)^ | 89.93 (± 6.64)  **^NS^**^(QBSL),^  **^NS^**^(QMSL)^ |  |
| Lower chest circumference (cm) | 83.01 (± 7.57)  **^NS^**^(QMSL),^  **^NS^**^(QBSL)^ | 84.50 (± 7.98)  **^NS^**^(QLHA),^  **^NS^**^(QBSL)^ | 86.15 (± 7.75)  **^NS^**^(QBSL),^  **^NS^**^(QMSL)^ |  |
| UCC to MCC (cm) | 10.32 (± 2.71)  **^NS^**^(QMSL),^  **^NS^**^(QBSL)^ | 9.63 (± 1.45)  **^NS^**^(QLHA),^  **^NS^**^(QBSL)^ | 9.93 (± 1.65)  **^NS^**^(QLHA),^  **^NS^**^(QMSL)^ |  |
| MCC to LCC (cm) | 7.31 (± 2.41)  **^NS^**^(QMSL),^  ******^(QBSL)^ | 7.69 (± 1.81)  **^NS^**^(QLHA),^  *****^(QBSL)^ | 8.98 (± 2.77)  ******^(QLHA),^  *****^(QMSL)^ |  |
| UCC to LCC (cm) | 17.63 (± 4.63)  **^NS^**^(QMSL),^  **^NS^**^(QBSL)^ | 17.32 (± 2.43)  **^NS^**^(QLHA),^  **^NS^**^(QBSL)^ | 18.91 (± 3.89)  **^NS^**^(QBSL),^  **^NS^**^(QMSL)^ |  |
| Resting Chest Volume (L) | 11.05 (± 2.71)  **^NS^**^(QMSL),^  **^NS^**^(QBSL),^  ***^(LSL)^ | 11.17 (± 2.17)  **^NS^**^(QLHA),^  **^NS^**^(QBSL),^  ***^( LSL)^ | 12.53 (± 2.90)  **^NS^**^(QLHA),^  **^NS^**^(QMSL),^  ***^( LSL)^ | 17.44 (± 2.36)  *******^(QLHA),^  *******^(QMSL),^  *******^(QBSL)^ |

**Table S1.** Full participant anthropometrics, with values given as mean ± SD. NS: not significant; *: p ≤ 0.05; **: p ≤ 0.01; ***: p ≤ 0.001.

**(A)**

**
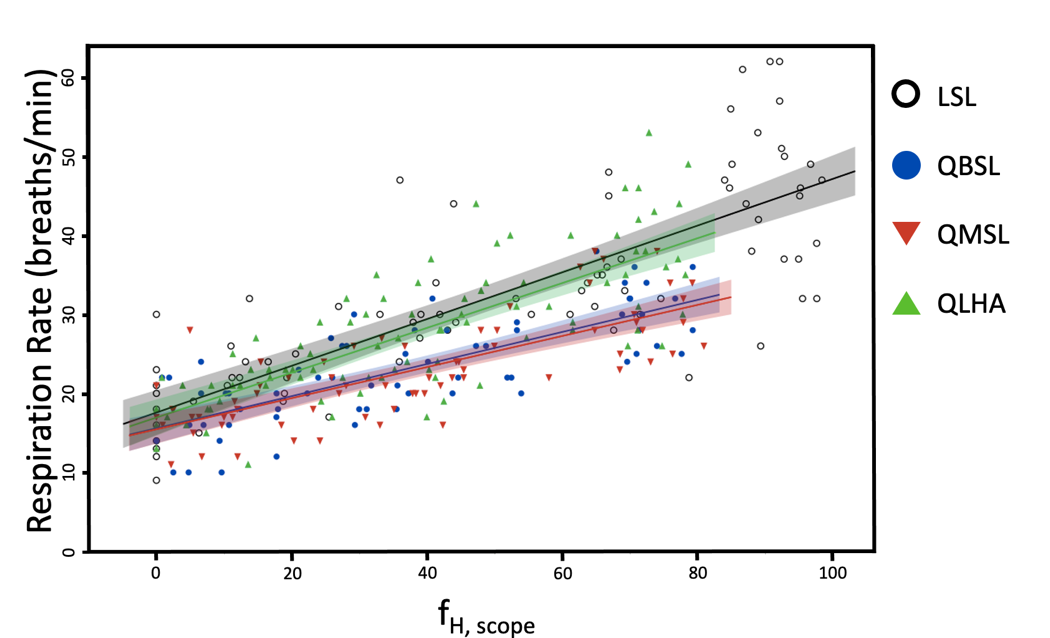
**

**(B)**

**
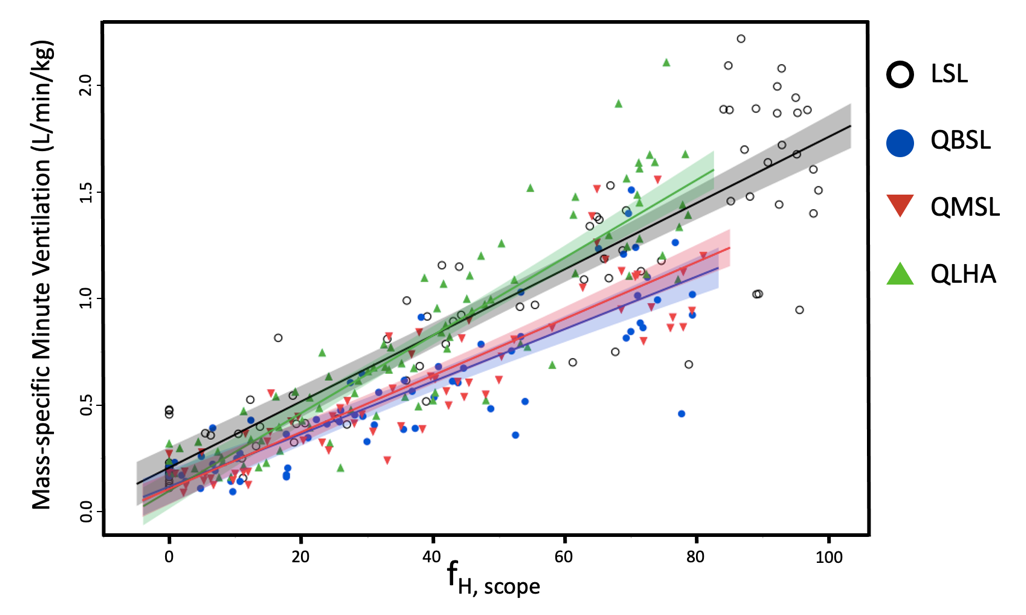
**

**Figure S4.** **(A)** f_R_ increases with f_H_ and V̇O_2_ across populations. **(B)** Mass-specific V̇_E_ also increases with f_H_ and V̇O_2_ across populations. LSL (slope ± S. 0.015 ± 0.001, R^2^=0.82; QLHA p<0.001; QMSL p=0.165; QBSL p=0.030) and QLHA (slope ± S. 0.018 ± 0.001, R^2^=0.82; QMSL p<0.001; QBSL p<0.001) increase minute ventilation more with increasing f_H_ than Quechua populations living at sea level. This is likely due physiological adaptations that allow Quechua at sea-level to better extract oxygen from inhaled air, thus not needing as much air as LSL at a given f_H_. QMSL (slope ± S. 0.013 ± 0.001, R^2^=0.80) and QBSL (slope ± S. 0.013 ± 0.001, R^2^=0.75) did not differ significantly (p=0.43).

**(A)**

**
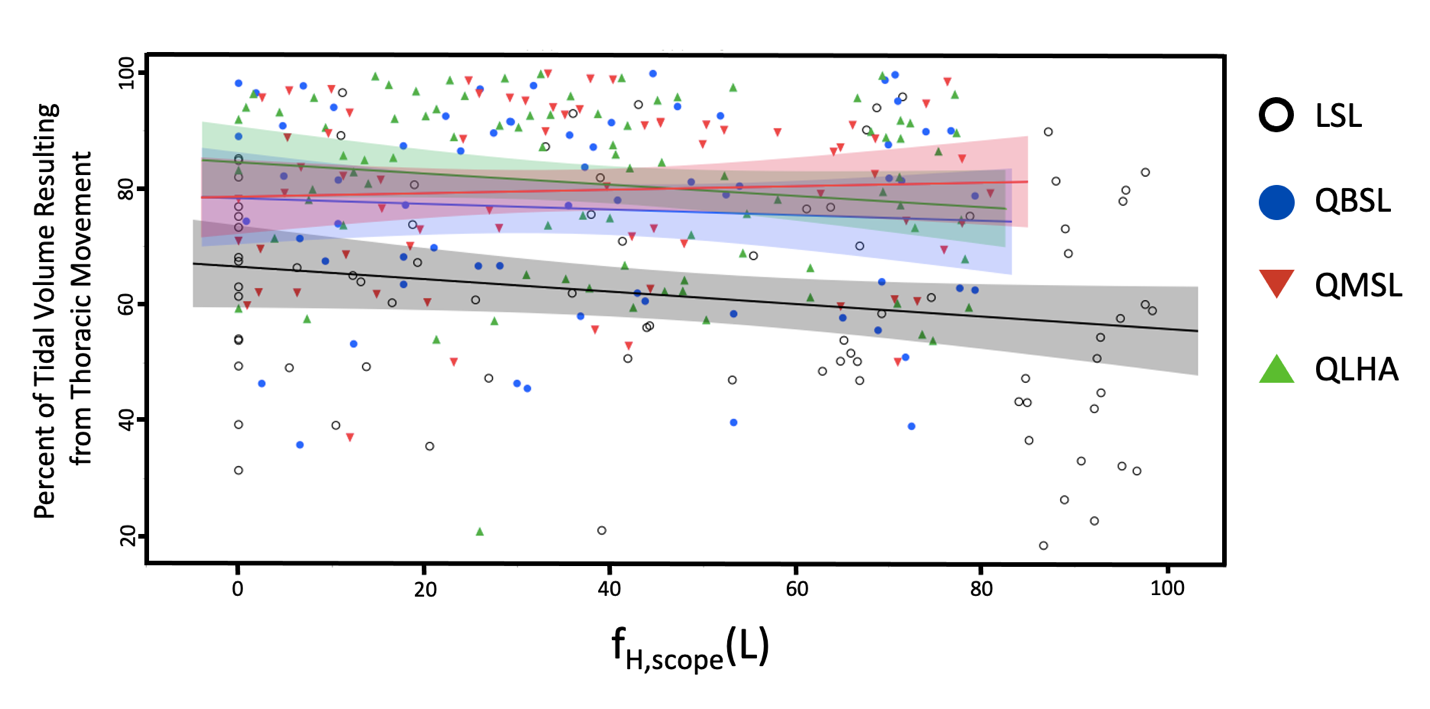
**

**(B)**

**
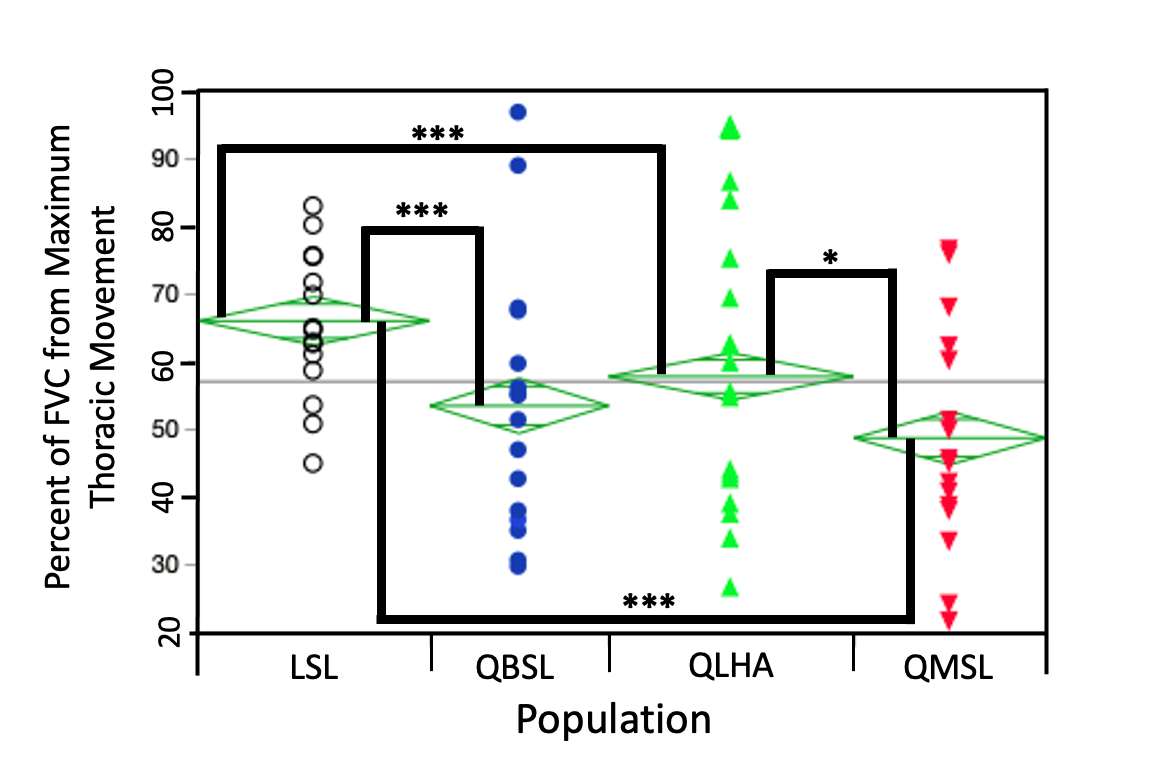
**

**Figure S5. (A)** Percent of tidal volume resulting from thoracic movement remained nearly constant across increasing heart rates when walking and running in all populations, with no significant changes between Quechua and LSL participants. However, Quechua participants did generally use more thoracic motion than LSL participants to breathe when active. **(B)** Quechua participants used the diaphragm to ventilate more than LSL participants during FVC.


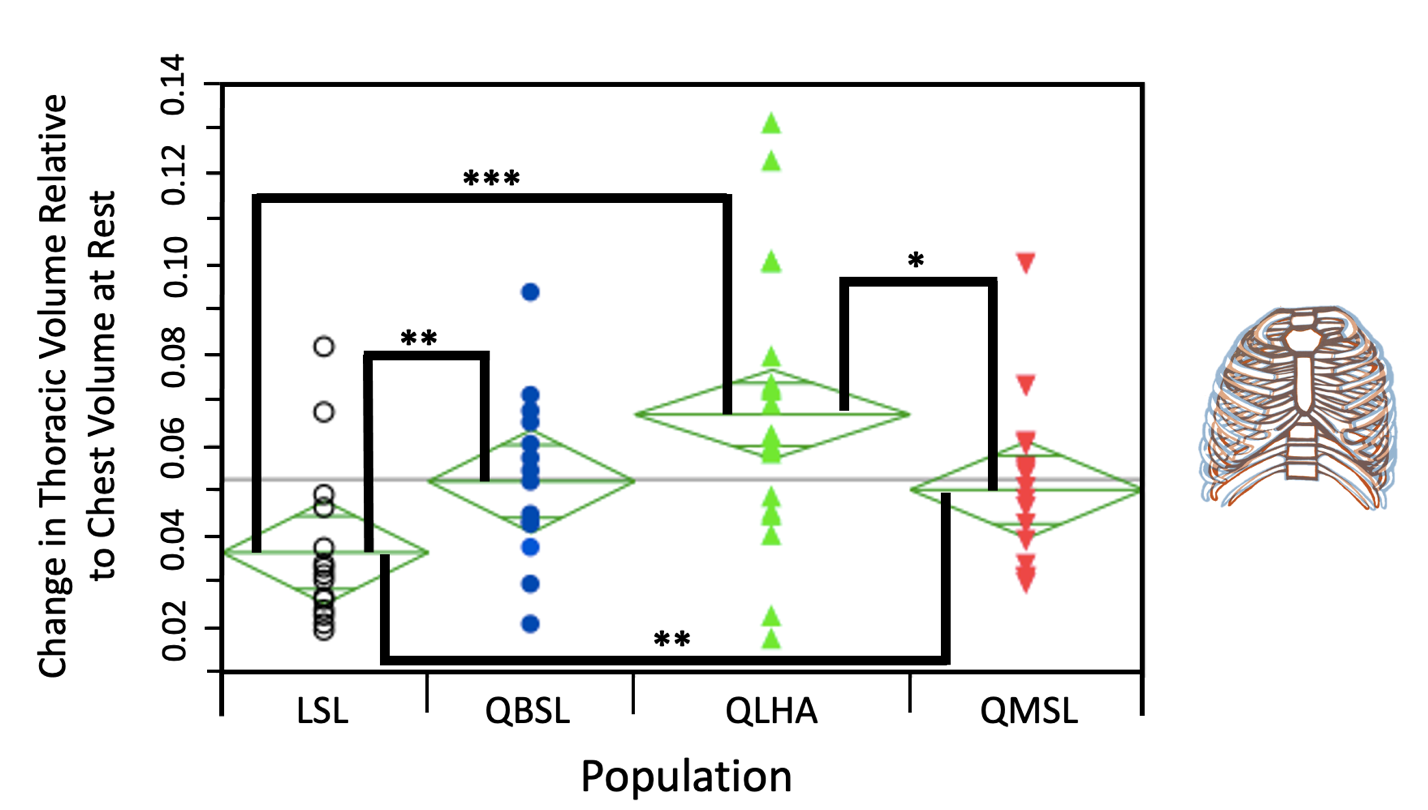


**Figure S6.** Change in thoracic volume per breath relative to chest volume at rest. Change in thoracic volume per breath relative to chest volume at resting f_H_ is greatest in QLHA participants. Differences were observed in resting thoracic volume change relative to chest volume between QLHA (mean ± s.e.m. 0.067 ± 0.005; 95% CI [0.057, 0.077]), QMSL (0.050 ± 0.005; 95% CI [0.039, 0.061]; p=0.03) and QBSL (0.052 ± 0.005; 95% CI [0.042, 0.062]; p=0.05) participants. The largest resting difference was observed between LSL participants (0.036 ± 0.006; 95% CI [0.025, 0.048]) and QLHA (p<0.001).

**
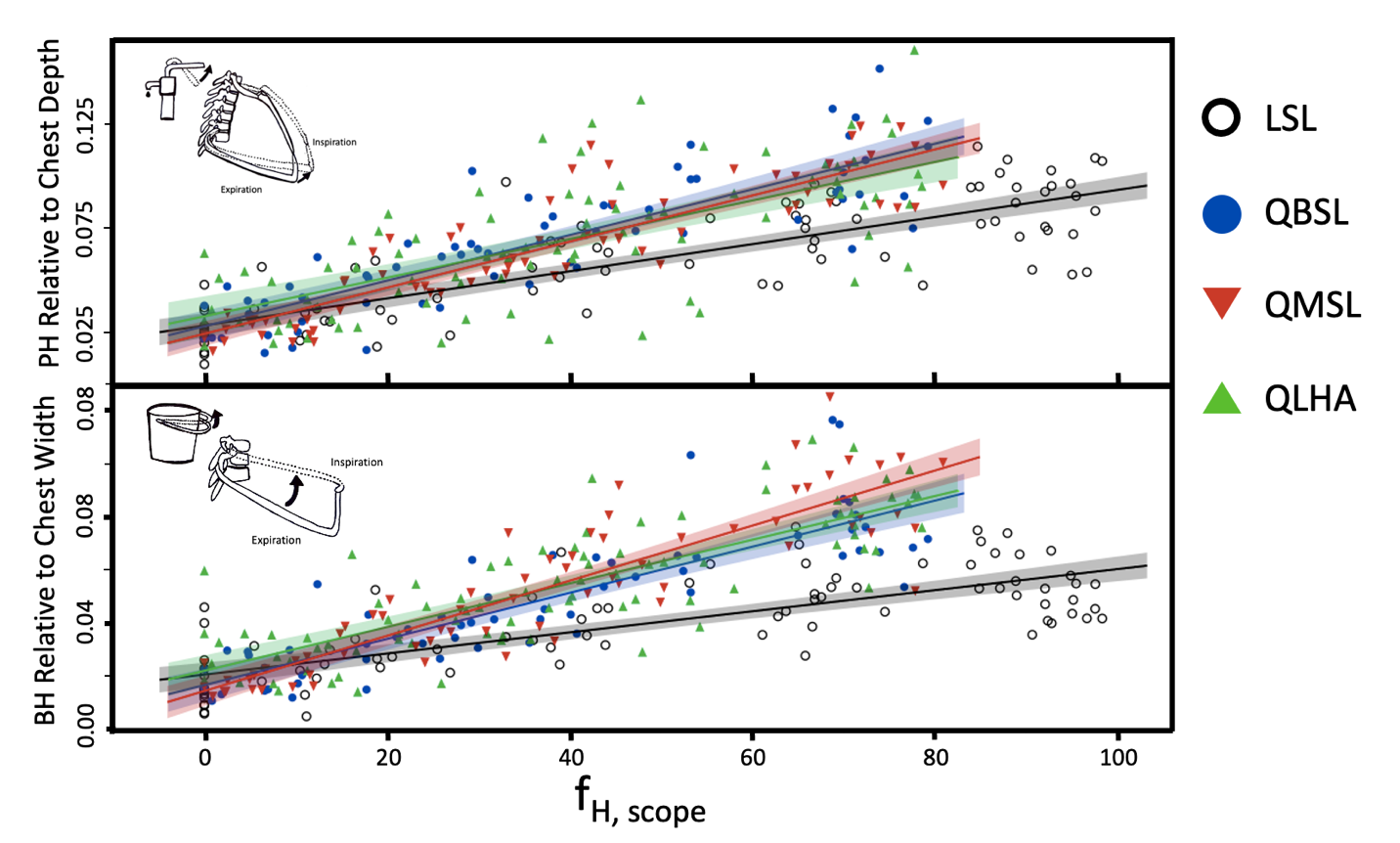
**

**Figure S7.** Mediolateral (BH) and dorsoventral (PH) thoracic expansions relative to heart rate. Dorsoventral expansion relative to chest depth increased significantly less with f_H_ in LSL participants (slope ± S. 0.00065 ± 0.00005, R^2^=0.73) than in QLHA (slope ± S. 0.00090 ± 0.00011, R^2^=0.45; p=0.037), QMSL (slope ± S. 0.00110 ±0.00007, R^2^=0.80; p<0.001) and QBSL (slope ± S. 0.00109 ± 0.00008, R^2^=0.73; p<0.001) participants. Changes in dorsoventral expansion relative to chest depth with increasing f_H_ were not significantly different between Quechua populations. Mediolateral expansion relative to chest width increased significantly less with f_H_ in LSL participants (slope ± S. 0.00033 ± 0.00004, R^2^=0.50) than in QLHA (slope ± S. 0.00078 ± 0.00007, R^2^=0.73; p<0.001), QMSL (slope ± S. 0.00103 ± 0.00006, R^2^=0.79; p<0.001) and QBSL (slope ± S. 0.00087 ± 0.00007, R^2^=0.73; p<0.001). Changes in mediolateral expansion relative to chest width with increasing f_H_ were not significantly different between Quechua populations.

**
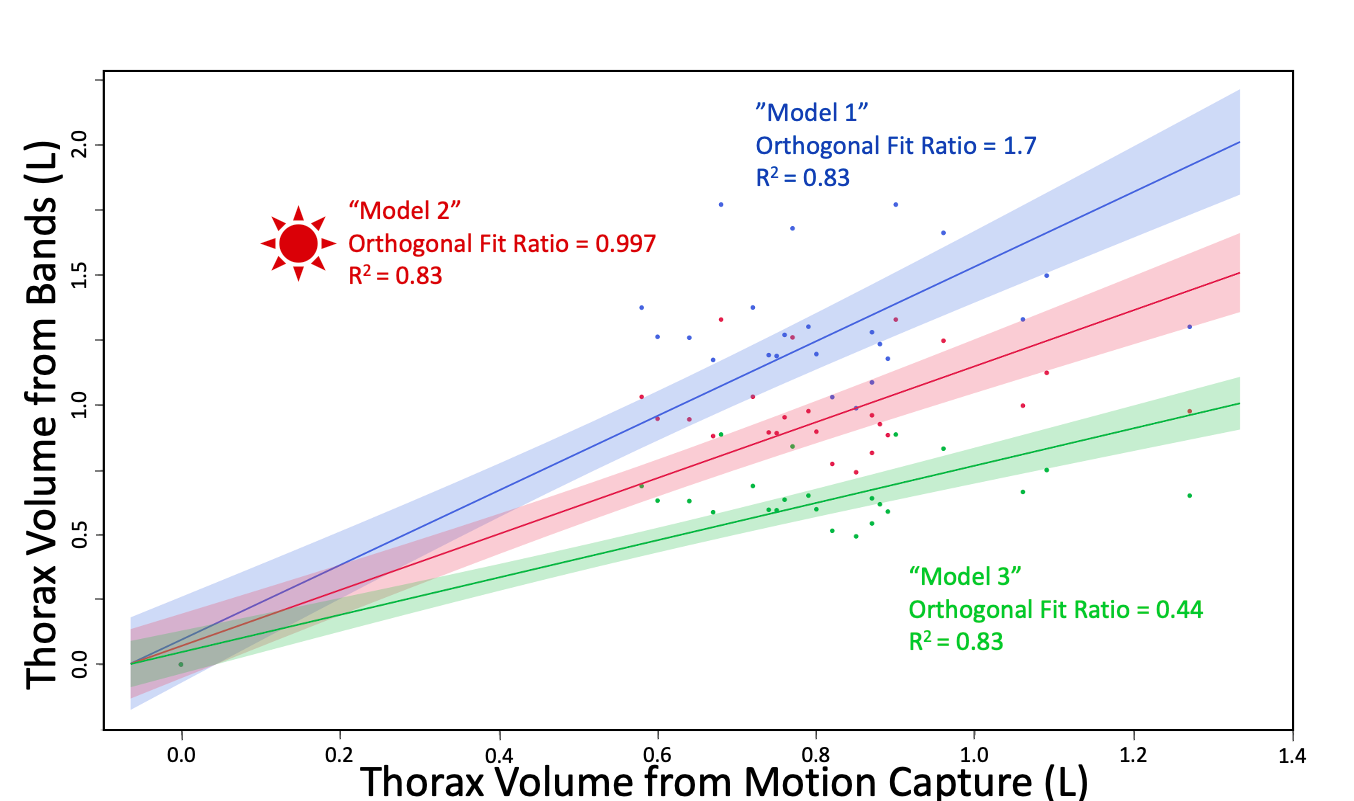
**

**Figure S8.** The overall thoracic volume was calculated through time using the devices (Banzett et al., 1995; Binks et al., 2007) and by modeling the thorax as a multi-part frustum (adapted from Ruff 1991; Fig. S10). Participants in a lab setting were outfitted with both our nanocomposite devices and reflective markers for motion capture (after Callison et al. 2019). Tidal volume (V_T_) was measured using a spirometer. Participants performed respiratory maneuvers while standing. Participants breathed quietly and deeply over the course of ten breaths each. While resting, participants also breathed while emphasizing rib cage displacement, followed by abdominal displacement after Banzett et al. (1995) – the same protocol used when taking measurements in Quechua participants in Lima, Peru and Cerro de Pasco, Peru away from the lab. Thoracic expansion was also measured during walking and running using bands and motion capture. Based on band measurements, three sperate models of thoracic volume change were calculated from the formula:

$$Volume=K \left( \frac{}{}UCC+ \frac{}{}MCC+LCC \right)$$

where $\frac{}{}$ and $\frac{}{}$ are dimensionless numbers representing the ratio of different levels of the ribcage and κ is a participant-specific constant that relates the proportioned and summed rib cage signals (measured from the three nanocomposite chest bands) to volume (adapted from Banzett et al. 1995). Following Banzett et al. (1995), data from ten breaths of quiet breathing and breathing with rib cage emphasis were used to calculate α, β, γ, λ, and κ for each participant with spirometric volume as a dependent variable. UCC, MCC, and LCC rib cage signals were measured at as a function of the measure spirometric volume.

Model 2 was the most reliably accurate across all thoracic volumes compared to validated 3D motion capture methods (Callison et al. 2019) with a high orthogonal fit ratio of 0.997. Additionally, two other models were used to estimate volumetric change in the thorax. Model 1 was found to be most accurate for the largest volumetric changes. Model 3 was found to conservatively underestimate thoracic volume change across all volumes.

Model 2 was selected for analysis of thoracic volume change in this paper due to its high accuracy across all measurements. Regardless, even the most conservative estimates of thoracic expansion and contraction during ventilation obtained using our nanocomposite devices (based on Model 3) suggest significant differences between high-altitude adapted Quechua populations and LSL participants. As such, we are confident in our findings that greater thoracic volume change is measured in Quechua. Our PH and BH measurements (Fig. S9) support with this conclusion.


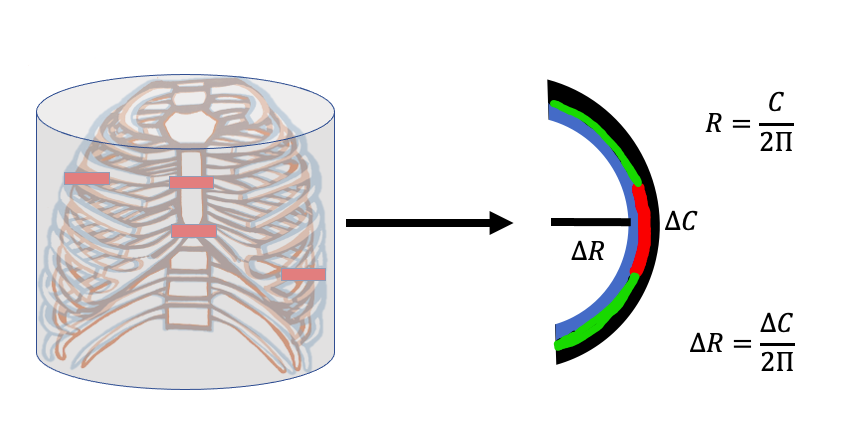


**Figure S9**. Method of calculating dorsoventral and mediolateral expansion.


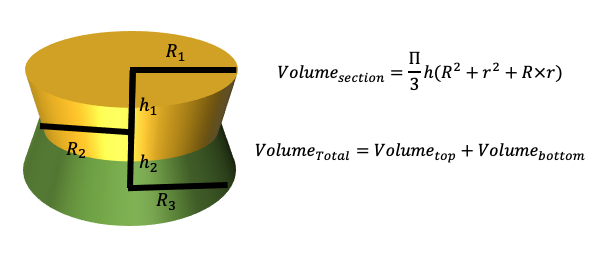


**Figure S10**. Model of the thorax as a multi-part, truncated cone.
